# Disruption of the homeodomain transcription factor *orthopedia homeobox* (*Otp*) is associated with obesity and anxiety

**DOI:** 10.1016/j.molmet.2017.08.006

**Published:** 2017-08-24

**Authors:** Lee Moir, Elena G. Bochukova, Rebecca Dumbell, Gareth Banks, Rasneer S. Bains, Patrick M. Nolan, Cheryl Scudamore, Michelle Simon, Kimberly A. Watson, Julia Keogh, Elana Henning, Audrey Hendricks, Stephen O'Rahilly, Inês Barroso, UK10K consortium, Adrienne E. Sullivan, David C. Bersten, Murray L. Whitelaw, Susan Kirsch, Elizabeth Bentley, I. Sadaf Farooqi, Roger D. Cox

**Affiliations:** 1 MRC Harwell Institute, Mammalian Genetics Unit and Mary Lyon Centre, Harwell Campus, Oxfordshire, OX11 0RD, UK; 2 University of Cambridge Metabolic Research Laboratories and NIHR Cambridge Biomedical Research Centre, Wellcome Trust-MRC Institute of Metabolic Science, Box 289, Addenbrooke's Hospital, Cambridge CB2 0QQ, UK; 3 The Blizard Institute, Barts and The London School of Medicine and Dentistry, Queen Mary University of London, London E1 2AT, UK; 4 School of Biological Sciences, University of Reading, Reading, Berkshire, UK; 5 Wellcome Trust Sanger Institute, Cambridge, UK; 6 Department of Mathematical and Statistical Sciences, University of Colorado-Denver, Denver, CO 80204, USA; 7 Department Molecular and Cellular Biology, University of Adelaide, Adelaide, Australia; 8 Department of Endocrinology, Hospital for Sick Children, 555 University Avenue, Toronto, Ontario, Canada

**Keywords:** OTP, Obesity, Energy balance, Mouse model, Human mutation, Oxytocin, Vasopressin

## Abstract

**Objective:**

Genetic studies in obese rodents and humans can provide novel insights into the mechanisms involved in energy homeostasis.

**Methods:**

In this study, we genetically mapped the chromosomal region underlying the development of severe obesity in a mouse line identified as part of a dominant N-ethyl-N-nitrosourea (ENU) mutagenesis screen. We characterized the metabolic and behavioral phenotype of obese mutant mice and examined changes in hypothalamic gene expression. In humans, we examined genetic data from people with severe early onset obesity.

**Results:**

We identified an obese mouse heterozygous for a missense mutation (pR108W) in *orthopedia homeobox* (*Otp*), a homeodomain containing transcription factor required for the development of neuroendocrine cell lineages in the hypothalamus, a region of the brain important in the regulation of energy homeostasis. *Otp*^*R108W/+*^ mice exhibit increased food intake, weight gain, and anxiety when in novel environments or singly housed, phenotypes that may be partially explained by reduced hypothalamic expression of oxytocin and arginine vasopressin. R108W affects the highly conserved homeodomain, impairs DNA binding, and alters transcriptional activity in cells. We sequenced *OTP* in 2548 people with severe early-onset obesity and found a rare heterozygous loss of function variant in the homeodomain (Q153R) in a patient who also had features of attention deficit disorder.

**Conclusions:**

OTP is involved in mammalian energy homeostasis and behavior and appears to be necessary for the development of hypothalamic neural circuits. Further studies will be needed to investigate the contribution of rare variants in OTP to human energy homeostasis.

## Highlights

• A mouse *Otp* mutation alters hypothalamic neuropeptide expression leading to increased food intake, obesity and anxiety.
• In severe early onset obesity, we found a heterozygous LOF variant in a patient with attention deficit disorder features.
• These studies show for the first time that mutations in the *Otp/OTP* gene cause obesity.

## 1 Introduction

The development of neuroendocrine cell lineages in the hypothalamus requires a number of transcription factors including *orthopedia homeobox* (*OTP*) and *single-minded-homology 1* (*SIM1*). With the exception of the arcuate nucleus (ARC), where *SIM1* is not found, *OTP* and *SIM1* are co-expressed at the same time and in the same cells [1]. Germline haploinsufficiency of *Sim1* in mice and loss of function mutations in *SIM1* in humans result in hyperphagic obesity [2], [3], [4], [5], [6]. Loss of *Sim1* in mice leads to perinatal death, but haploinsufficient mice are viable and show a reduction in the number of arginine vasopressin (AVP) and oxytocin (OXT) producing neurons in the paraventricular nucleus (PVN) [7]. Disruption of Sim1 expression in adult mice leads to increased food intake and obesity, suggesting that Sim1's effects on energy homeostasis are not confined to its role in hypothalamic development [8].

In contrast to *SIM1*, there have been no reports to date of obesity associated with *OTP* mutations or haploinsufficiency. OTP is expressed in neurons of the PVN, supraoptic (SON), anterior periventricular (aPV), and ARC and during development in the neural tube and hindbrain [1], [9], [10]. The Genotype-Tissue Expression, GTEx portal (https://www.gtexportal.org/home/gene/OTP on 21/07/2017) reports little expression outside of the hypothalamus. Homozygous knockout *Otp* mice are perinatal lethal and show reduced cell proliferation, abnormal cell migration and defective aPVN, PVN, ARC, and supraoptic (SON) nuclei-neuronal differentiation. Thus, the *Otp* gene regulates the fate, migration, and terminal differentiation of hypothalamic neurons. Further, previous studies in flies, zebrafish, and mice have shown that *Otp* modulates the development of neuroendocrine cell lineages expressing somatostatin (*Sst*) and thyrotropin-releasing hormone (*Trh*) [1], [9], [11], [12], [13], [14], [15], and, acting with *Sim1*, regulates the development of *Oxt*, *Avp*, and corticotrophin-releasing hormone (*Crh*) expressing neurons in the hypothalamus [1], [9], [16], [17].

In the present study, we have carried out a high throughput dominant N-ethyl-N-nitrosourea (ENU) mutagenesis screen in mice with the objective of identifying novel obesity models. We report here an obese model with a novel hypomorphic mutation in the orthopedia homeobox (*Otp*) gene.

## 2 Materials and methods

Full methods and experimental details are described in Appendix A and brief descriptions are provided below.

### 2.1 Mouse studies

Animal studies were performed under guidance issued by the Medical Research Council (MRC) in Responsibility in the Use of Animals for Medical Research (July 1993) and UK Home Office Project Licences 30/3146 and 30/3384. Mouse *Otp* mutation cohorts were maintained on a C3H/HeH background (C3H/HeH-OtpR108W). For complementation tests *Otp*^*R108W/+*^ were maintained by backcross to C57BL/6J. *Otp*^*tm1Asim/Cnrm*^ mice were generated as previously described [1] and derived onto a C57BL/6J background. Genome mapping was carried out using a KBioscience panel of 91 SNP's informative between C57BL/6J and C3H/HeH. Sequence alignment was carried out using CLUSTAL W (1.81) multiple sequence alignment in ENSEMBL GRCm38, version 75.

### 2.2 Mouse phenotyping

IPGTTs were carried out according to the IMPReSS protocols (www.mousephenotype.org/impress/protocol/87/8). Intraperitoneal insulin tolerance tests (ipITT) were carried out on animals fasted for 5–6 h and blood sampled under a local anesthetic at 0 min (baseline), prior to injection of 1.5 IU insulin per kg of body weight, and subsequently at 15, 30, 45, 60, and 90 min. To measure food intake, male mice of each genotype, wild-type and heterozygote, were housed in pairs of the same sex and genotype from 4 weeks of age until the experiment concluded [18]. Body composition was determined by quantitative NMR (Echo MRI, Echo Medical System, Houston, TX). Dual-energy X-ray Absorptiometry (DEXA, PIXImus, Wisconsin, USA) was used to quantify terminal fat mass, lean mass, and bone mineral content and density. Fecal 24 h samples were dried at 55 °C for 48 h and ∼1 g was burnt in a bomb calorimeter (IKA^®^ C2000 Basic Calorimeter, Staufen, Germany) for determination of energy content. Metabolic rate was measured using indirect calorimetry (Oxymax, Columbus Instruments). Adjustment of VO_2_, VCO_2_, and Energy Expenditure (EE) for variation in lean mass was by multiple linear regression analysis (ANCOVA) as outlined in McMurray et al., [19]. Full details of the open field analysis are given on the Impress database (www.mousephenotype.org/impress). Home cage activity monitoring of group housed animals was as described in [20]. In the Light/Dark box, individual mice were placed into one corner of an enclosed arena (overall dimensions 40 cm × 40 cm) and allowed to explore for 20 min while monitored by EthoVision XT analysis software (Noldus).

### 2.3 RNA and protein analysis

RNA was extracted from snap frozen tissues collected between 2 and 3 h after lights on from free fed mice using an RNeasy Mini Kit (Qiagen, UK). Samples were analyzed in triplicate using TaqMan probes and gene expression normalized relative to house-keeping genes as described in Appendix A. Total protein was extracted from the hypothalamus of P0 *Otp*^*+/+*^, *Otp*^*R108W/+*^, *Otp*^*+/tm1Asim*^ and *Otp*^*R108W/tm1Asim*^ mice. Western blotting and antibodies are as described in Appendix A. For immunohistochemical analysis, adult mice (10 weeks old) brains were collected at approximately 7 h after lights on, immediately immersed in cold 4% paraformaldehyde (PFA)-PBS, and incubated at 4 °C for 24 h and then in 30% sucrose-PBS at 4 °C until sinking. Coronal cryosections were cut at 10 μm and collected on charged slides. Sections were probed with antibodies as described in Appendix A.

### 2.4 Human studies

Ethical approval for studies was given by the Cambridge Research Ethics Committee; all participants gave written informed consent. The Genetics of Obesity Study (GOOS) is a cohort of 7000 individuals with severe early-onset obesity; age of obesity onset is less than 10 years. Severe obesity is defined as a body mass index (weight in kilograms divided by the square of the height in meters) standard deviation score greater than 3 (standard deviation scores calculated according to the United Kingdom reference population). Targeted Sequencing (TS) and Whole Exome Sequencing (WES) were performed as described as part of the UK10K consortium [21].

### 2.5 Reporter assays

Human cDNA of human OTP with C-terminal 3XFlag tags and respective gene variants were synthesized and cloned into pcDNA5/Frt/TO (Invitrogen), and Luciferase reporter constructs 8xnp and 6xP3 were synthesized and cloned into vector pLuc-MCS (Stratagene) by GenScript (NJ, USA). HEK293-T cells were transfected in triplicate and assayed as described in Appendix A.

### 2.6 Electrophoretic mobility shift assays

Wt or variant OTP-3xFlag expression vectors were transfected into HEK293-T cells and after 72 h nuclear extracts prepared as described in Appendix A. Nuclear extracts (30 μg of protein) were incubated with 40 nM 6-FAM 5′ labeled oligo (P3-gcaccTAATCCGATTAgcacc or NP-gcgTCAATTAAATgcg) and 0.1 μg/μl of poly [d(I–C)] and DNA-protein complexes analyzed on a 7% polyacrylamide gel and imaged using a Chemidoc Digital Imager (Bio-Rad) as describe in Appendix A.

### 2.7 Cell imaging

Transfected HEK-293T cells were incubated for 24 h in growth medium and then fixed using 4% formaldehyde and permeabilized in 0.1% Triton X100, followed by immunostaining with monoclonal Flag M2 antibody (Sigma) diluted to 1:100, with secondary α-mouse Alexa Fluor 568 (Thermo Fisher Scientific) and DAPI (Thermo Fisher Scientific) stained. Cells were visualized with Confocal Laser Scanning Platform Leica TCS SP8 microscope (Leica Microsystems). Individual images were processed and analyzed using ImageJ software (http://imagej.nih.gov/ij/).

## 3 Results

### 3.1 Obesity in a mutagenized mouse model maps to the *Otp* gene

**Figure 1:**
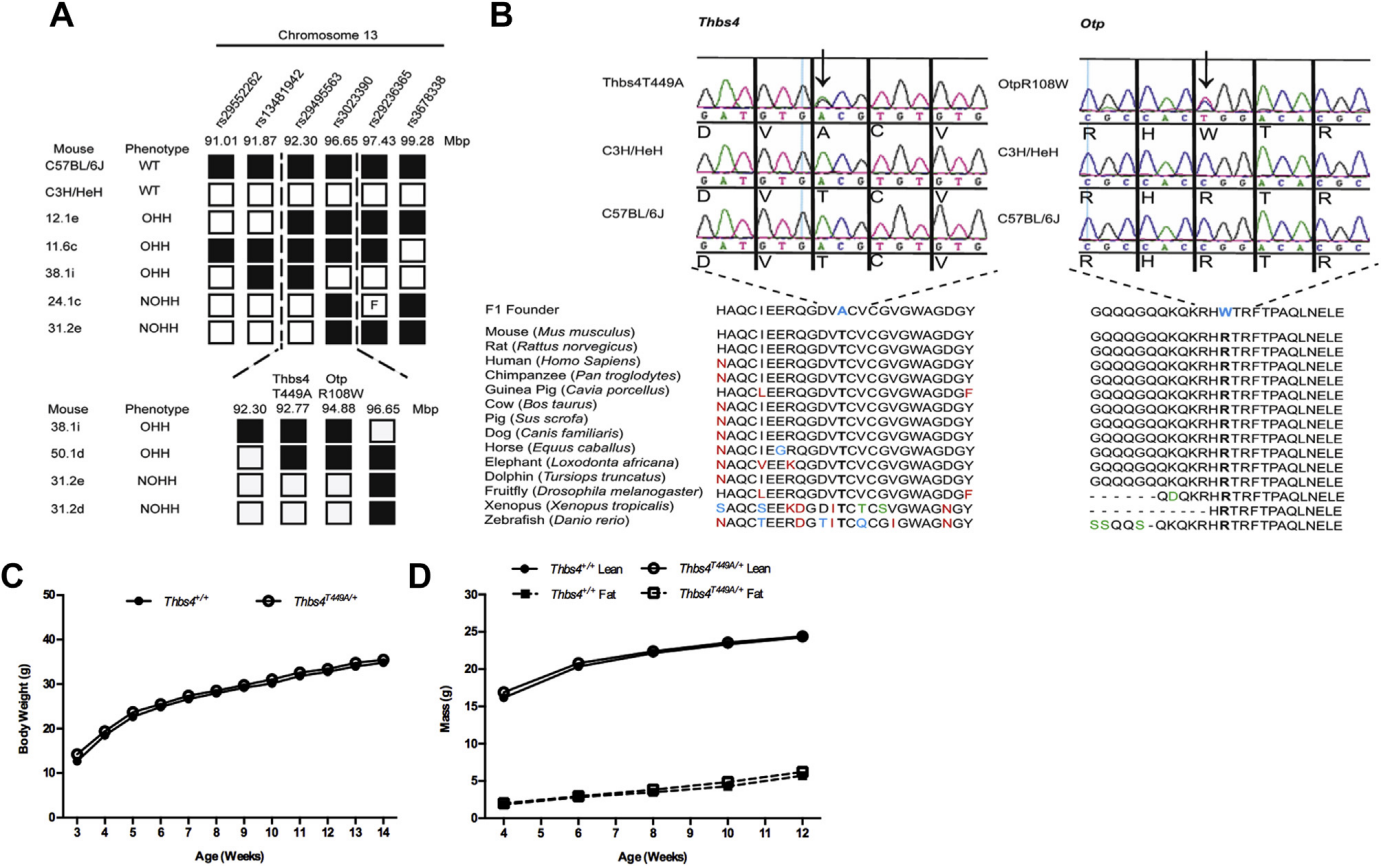
**Mapping and identification of the *Otp***^***R108W/+***^**mutation**. (**A**) Mapping and haplotype analysis identified a region of 4.4 Mbp on chromosome 13 (ENSEMBL GRCM38, version 87); OHH are obese, hyperglycemic, and hyperinsulinemic; NOHH are non-obese, non-hyperglycemic, and non-hyperinsulinemic. (**B**) Validation of *Thbs4* and *Otp* mutations in the F1 founder. Aligned amino acids in black, red, green, and blue are completely conserved residues, a different residue but with function conserved, a different residue but with function semi-conserved and a different residue with no conservation of function, respectively. (**C and D**) Exclusion of *Thbs4*^*T449A/+*^ as the causal gene in a cohort carrying *Thbs4*^*T449A/+*^ and wildtype *Otp*. Weekly body weights (**C**) and fortnightly EchoMRI Lean mass (solid line) and fat mass (dotted line) mean ± SEM (**D**) of *Thbs4*^*T449A*^ male mice showed no phenotype. For (**C** and **D**) *Thbs4*^*+/+*^ n = 23–24, *Thbs4*^*T449A/+*^ n = 23–24.

**Figure 2:**
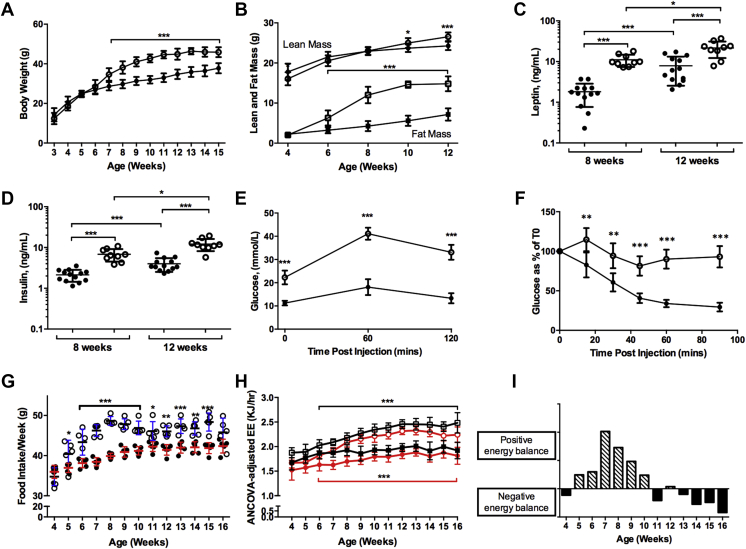
**Male *Otp***^***R108W/+***^**mutation mice are obese, glucose intolerant and insulin resistant**. (**A**), Bodyweights. (**B**), Fat (square) and lean (circle) mass. (**C**), Plasma leptin after a 4–6 h fast at 8 and 12 weeks. (**D**), Plasma insulin concentrations after a 4–6 h fast at 8 and 12 weeks. (**E**), Intraperitoneal Glucose Tolerance Test (IPGTT) at 12 weeks after a 5–6 h fast. (**F**), Intraperitoneal insulin sensitivity test (ipITT) at 12 weeks. (**G**), Food intake. (**H**), ANCOVA-adjusted EE in light (red lines) and dark (black lines) respectively. (**I**) Calculated energy balance. For (**A**–**H**) *Otp*^*R108W/+*^ data are shown as open symbols, *Otp*^*+/+*^ are filled symbols and in (**B**) circles are lean mass and squares fat mass. (**A**–**D**) *Otp*^*R108W/+*^ n = 9 and *Otp*^*+/+*^ n = 13, for (**E**) *Otp*^*R108W/+*^ n = 7 and *Otp*^*+/+*^ n = 13, for (**F**) *Otp*^*R108W/+*^ n = 7 and *Otp*^*+/+*^ n = 8, for (**G**) *Otp*^*R108W/+*^ n = 5 (pairs of mice) and *Otp*^*+/+*^ n = 5 (pairs of mice), for (**H**) *Otp*^*R108W/+*^ n = 10 and *Otp*^*+/+*^ n = 8 to 10. For (**A**, **E**, **G**) data were analyzed by 2-way Repeated Measures ANOVA with Bonferroni post-tests and, in addition, for (**E**) AUC was also calculated using individual t = 0 baselines and was increased for heterozygotes (p = <0.0001). For (**B**) AUCs were calculated and increased for both lean mass (p = 0.0026) and fat mass (<0.0001) in heterozygotes. Differences in AUCs and individual time points were tested with a Mann–Whitney 2-tailed t-test. For (**C**, **D**), an unpaired Mann–Whitney two-tailed t-test was used within age points between genotype classes and a Wilcoxon 2-tailed paired t test between time-points for the same genotype. For (**F**), AUC was calculated using 0% as a baseline and was increased in heterozygotes (p = 0.0003). Differences in AUCs and individual time points were tested with an unpaired Mann–Whitney 2-tailed t-test. For (**H**), data were normalized for lean mass and analyzed by ANCOVA. Mean ± SD. *p < 0.05, **p < 0.01, ***p < 0.001.

In this study, we identified a point mutation in *Otp* in an obese male mouse (61.4 g at 14-weeks) emerging from a high throughput dominant mouse *N*-ethyl-*N*-nitrosourea (ENU) mutagenesis screen. We confirmed the phenotype in 33 mice generated by backcrossing the F1 animal to C3H/HeH and mapped the genomic region underlying obesity to chromosome 13 (91.01 Mbp–99.28 Mbp), refining the interval to 92.3–96.65 Mbp with further backcrosses (Figure 1A). Using whole-genome sequencing, we identified four mutations that could contribute to the phenotype; Sanger sequencing of the F1 founder confirmed only two of these (Figure 1B). The first mutation was in the *Thbs4* gene at base 1490 (ENSMUST00000022213.7), which was mutated from an A (in the parental strains C3H/HeH and C57BL/6J) to a G in the F1 founder, resulting in a missense amino acid change at residue 449, threonine (T) to alanine (A), pT449A. The second mutation was in the *Otp* gene at base 490 in ENSMUST00000022195.11, which was mutated from a C to a T in the F1 founder, resulting in a missense amino acid substitution at residue 108, arginine (R) to tryptophan (W), pR108W. Both mutations are in highly conserved regions (Figure 1B). Backcrossing to produce a congenic line resulted in segregation of these mutations; *Thbs4* mutation carriers were not phenotypically different from wild type littermates (Figure 1C and D), in contrast to *Otp*^*R108W/+*^ mice which were obese (Figure 2A and B and Appendix B Supplementary Figure 1a and d).

### 3.2 Homozygous R108W mice show perinatal lethality, and heterozygous mice are obese

We found that mice homozygous for the *Otp* mutation died before weaning (Appendix C Supplementary Table S1); gross pathological changes were not seen (Appendix D). We performed a series of studies in heterozygous *Otp*^*R108W/+*^ mice (congenic at backcross >10) and WT littermates fed *ad libitum* on a chow diet. *Otp*^*R108W/+*^ mice were significantly heavier than wild-type colony mates from 7 weeks of age (p < 0.001) (Figure 2A, Appendix B Supplementary Figure 1a) due almost entirely to an increase in white adipose tissue mass (p < 0.001) (Figure 2B and Appendix B Supplementary Figure 1d). Fat depot weights were similarly increased and bone mineral density was reduced (Appendix C Supplementary Table 2). *Otp*^*R108W/+*^ mice were hyperleptinemic, consistent with their increased fat mass, and hyperinsulinemic with impaired glucose tolerance and insulin resistance (Figure 2C–F, Appendix B Supplementary Figure 1g,j,m,p). Heterozygotes were significantly longer (nose to base of tail) than wild type littermates (female and male *Otp*^*+/+*^ 10.46 ± 0.19 cm (n = 10), *Otp*^*R108W/+*^ 10.75 ± 0.29 cm (n = 10) and *Otp*^*+/+*^ 10.59 ± 0.25 cm (n = 10), *Otp*^*R108W/+*^ 10.97 ± 0.19 cm (n = 9), p = 0.0059 and 0.0029 (mean ± SE, Mann–Whitney 2-tailed t-test) respectively).

### 3.3 Obesity in *Otp*^*R108W/+*^ mice results from increased food intake resulting in net positive energy balance

To delineate the mechanisms underlying the obesity of *Otp*^*R108W/+*^ mice, we measured weekly food intake and energy expenditure (EE) between 4 and 16 weeks in male mice. We observed an increase in food intake in *Otp*^*R108W/+*^ mice from 5 to 15 weeks of age (Figure 2G). Resting (light) and active (dark) 24 h EE normalized by ANCOVA for lean mass was similar at 5 weeks but increased significantly in *Otp*^*R108W/+*^ mice from 6 weeks of age in parallel with weight gain (Figure 2H, Appendix B Supplementary Figure 2a and b (un-normalized data regressed against lean mass as an example of raw data at 8 weeks of age) and c–h **(**ANCOVA adjusted VO_2_ and VCO_2_ together with RER)). The calculated metabolizable energy intake (MEI) value takes into account the energy lost in feces and urine. While there was no difference in fecal energy content, MEI was significantly increased between 7 and 14 weeks of age, corresponding to the period of active weight gain relative to controls (Appendix B Supplementary Figure 2i and j). *Otp*^*R108W/+*^ mice extracted significantly more energy from their food (daily EI/MEI as a percentage) at 11 weeks of age (Appendix B Supplementary Figure 2k) and were in overall positive energy balance between 5 and 10 weeks leading to obesity (Figure 2I).

### 3.4 Compound heterozygous knockout and R108W allele mice do not show complementation

**Figure 3:**
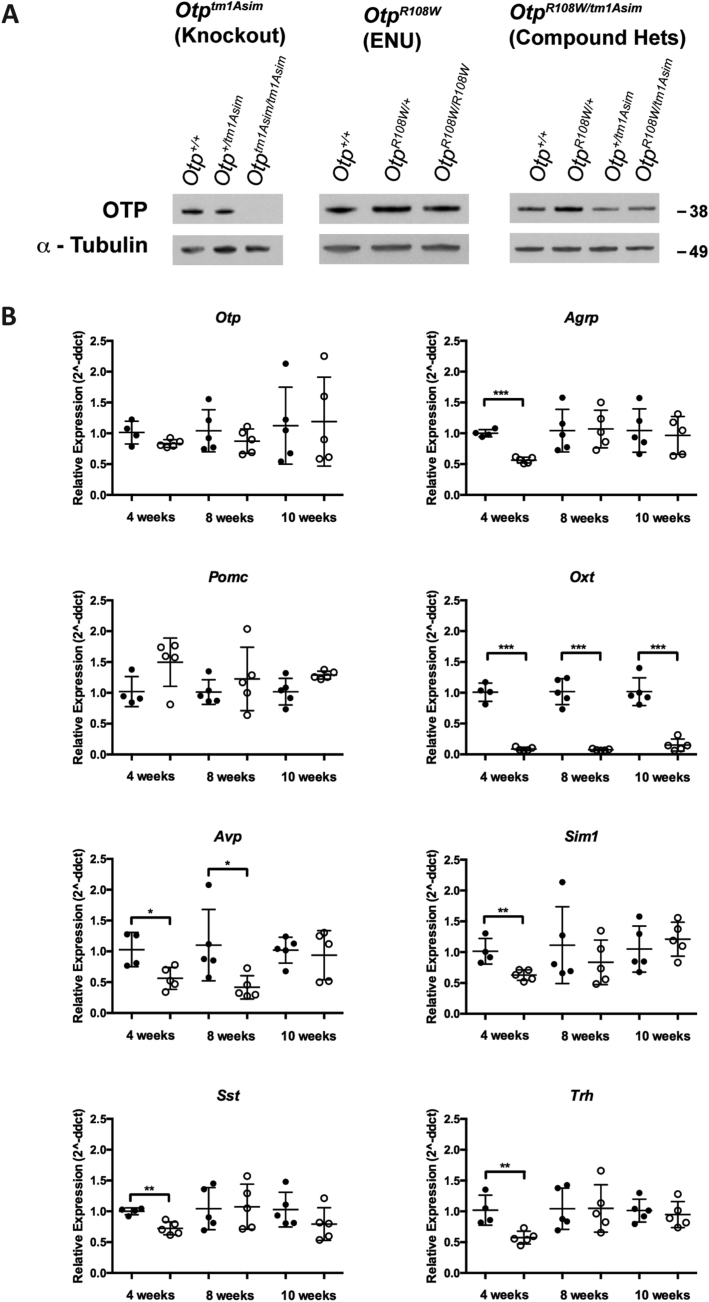
**Reduced expression of OTP, key hypothalamic and PVN neuronal markers**. (**A**), Representative western blot analysis of OTP and α-tubulin expression in the hypothalamus of *Otp*^*tm1Asim*^ (knockouts), *Otp*^*R108W*^ (ENU mutant), and *Otp*^*R108W/tm1Asim*^ (compound heterozygotes) mice. *Otp*^*tm1Asim*^ n = 5 biological replicates/genotype, *Otp*^*R108W*^ n = 2 biological replicates/genotype, *Otp*^*R108W/tm1Asim*^ n = 5 biological replicates per genotype. (**B**), Hypothalamic expression of key neuroendocrine genes by quantitative PCR analysis of RNA from male *Otp*^*+/+*^ (filled symbols) and *Otp*^*R108W/+*^ (open symbols) colony mates at 4, 8, and 10 weeks of age. Taqman assays were carried out in triplicate for each sample using probes for *Otp*, Agouti related peptide (*Agrp*), Pro-opiomelanocortin (*Pomc*), Oxytocin (*Oxt*), Arginine Vasopressin (*Avp*), Single-minded homolog 1 (*Sim1*), Somatostatin (*Sst*), and thyrotropin releasing hormone (*Trh*). Data expressed as mean ± SD for n = 5, except for wildtypes at 4 weeks where n = 4, analyzed with a two-tailed t-test, except for *Pomc* at 10 weeks, for which an unpaired Mann–Whitney 2-tailed t-test was used. Because of the low expression of *Oxt* in heterozygotes leading to unequal variance in comparison to the wildtypes, the data were log transformed at 4 and 8 weeks before application of the t-test. *p < 0.05, **p < 0.01, ***p < 0.001.

We next studied a published knockout *Otp* mouse line, *Otp*^*tm1Asim*^ that has not been reported to show altered body composition [1], [9]. After backcrossing onto C3H/HeH for three generations to allow comparison with *Otp*^*R108W/+*^ mice, *Otp*^*+/tm1Asim*^ animals showed a modest obesity phenotype (Appendix B Supplementary Figure 1b,c,e,f) and other similarities to Otp^*R108W*/+^ mice (Appendix B Supplementary Figure 1h,i,k,l,n,o,q,r). Crossing male *Otp*^*R108W/+*^ with female *Otp*^*+/tm1Asim*^ (on a C57BL/6J background), we generated compound heterozygotes that did not survive to weaning, indicating that the two mutations do not complement each other for postnatal lethality and demonstrating the functional consequences of the R108W mutation (Appendix C Supplementary Table 3). Hypothalamic *Otp* protein and mRNA levels were comparable in *Otp*^*R108W/+*^ mice compared to WT mice (Figure 3A and B); however, animals carrying both mutations (*Otp*^*R108W/tm1Asim*^) showed reduced *Otp* protein levels similar to *Otp*^*+/tm1Asim*^ KO heterozygotes (Figure 3A). Collectively, these data suggest that the *Otp*^*R108W/+*^ obesity causing allele is a loss of function mutation that potentially has a dominant negative effect leading to a more pronounced obesity phenotype than seen in the heterozygous null animal (Appendix B Supplementary Figure 1).

### 3.5 Altered expression of oxytocin and other neuropeptides in the hypothalamus of *Otp*^*R108W/+*^ mice

**Figure 4:**
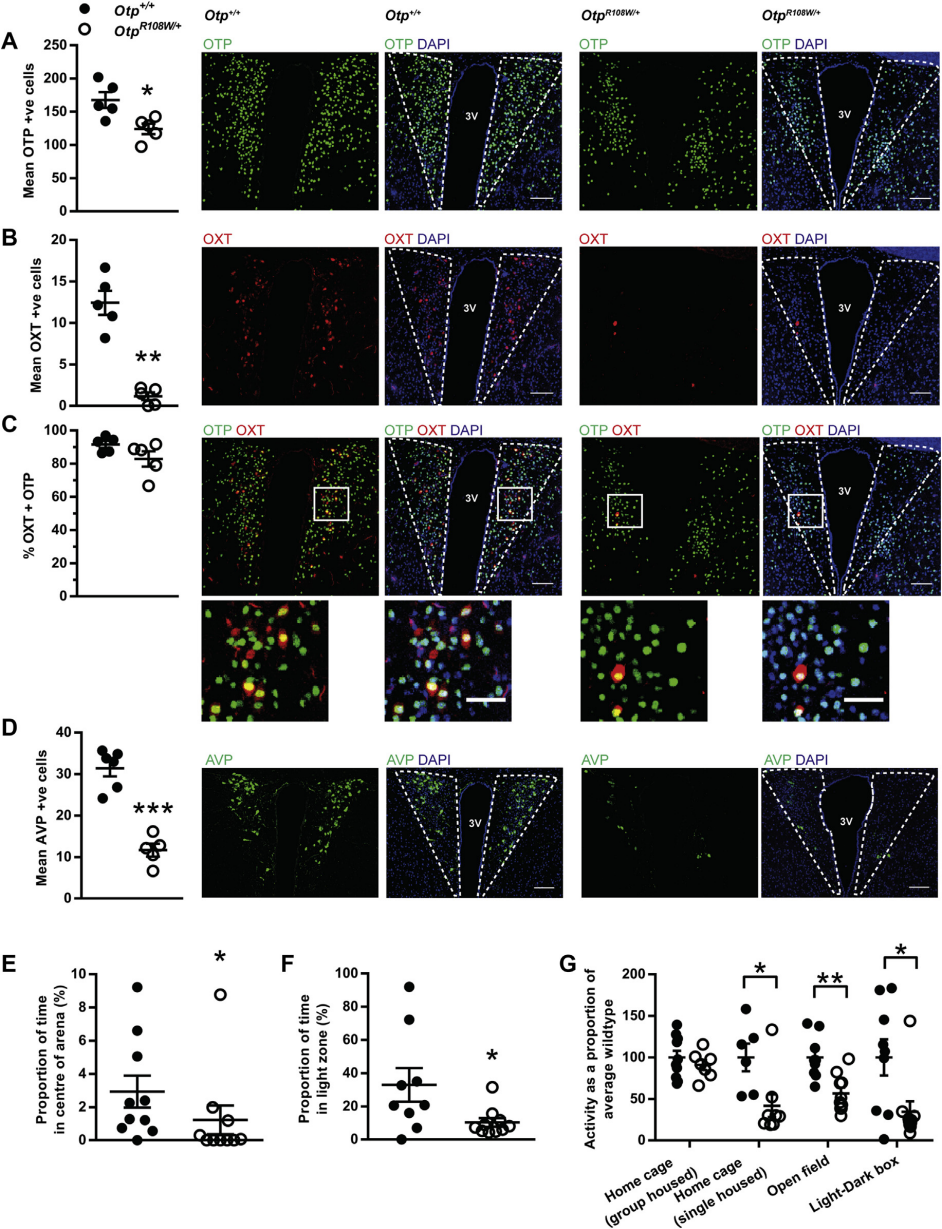
**Reduced expression of PVN neuronal markers.** The number of (**A**) OTP, (**B**) OXT (Oxytocin), (**C**) OTP and OXT, and (**D**) AVP (Arginine Vasopressin) expressing PVN neurons are significantly reduced in *Otp*^*R108W/+*^ (n = 5) compared to *Otp*^*+/+*^ (n = 5–6) mice, sacrificed at 10 weeks old. In neurons expressing OXT, the proportion of co-localization with OTP did not significantly differ between genotypes. Example images are shown for each genotype and staining combination, with and without DAPI nuclear stain. In (**C**), Co-localization image inserts are shown below, with corresponding region indicated by solid line box. Dashed lines indicate PVN, 3 V: third ventricle. Scale bars represent 100 μm for PVN images, 50 μm for inset images. **(E**) Open Field analysis *Otp*^*R108W/+*^ n = 10 and *Otp*^*+/+*^ n = 10. (**F**), Light–Dark Box analysis *Otp*^*R108W/+*^ n = 10 and *Otp*^*+/+*^ n = 9. (**G**), Locomotor activity comparison in Home cage (single and group housed), Open Field and Light–Dark box *Otp*^*R108W/+*^ n = 8, 7, 10, 10 and *Otp*^*+/+*^ n = 6, 10, 9, 9 respectively. For (**A, D and G**) unpaired two-tailed t-test and for (**B**, **C**, **E and F**) Mann–Whitney two-tailed t-test was used. Data expressed as mean ± SE *p < 0.05, ***p < 0.0001.

To explore the neural circuits affected by *Otp* disruption, we examined the hypothalamus of *Otp*^*R108W/+*^ mice. We found that agouti-related peptide (*Agrp*) mRNA levels were reduced at 4-weeks but normalized by 8- and 10-weeks, while pro-opiomelanocortin (*Pomc*) expression trended to elevation (p = 0.056 Mann–Whitney) at 10 weeks (Figure 3B), suggesting that the hyperphagia of these mice may be driven by mechanisms downstream of these signals; there were no differences in *Crh,* leptin receptor (*Lepr*), and melanocortin 4 receptor (*Mc4r*) expression (data not shown). We found a striking reduction in *Oxt* at all time points measured and *Avp* expression at 4 and 8 weeks (10–20% and 40–50% of wildtype levels respectively) (Figure 3B) and reduced *Sim1*, *Sst,* and *Trh* mRNA levels at 4 weeks (Figure 3B**),** consistent with a role for *Otp* in the development of these neuronal populations. Interestingly, SST is involved in inhibiting growth hormone release (reviewed [22]), and therefore the transient lower mRNA expression of *Sst* may help explain the small increase in body length of mutants. Notably, we observed a reduction in the number of OTP, OXT, and AVP positive staining cells in the PVN where oxytocin neurons are thought to be involved in regulating feeding behavior through projections to the nucleus tractus solitarius [23], [24], [25] (Figure 4A–D). Serum thyroxine was significantly reduced in *Otp*^*R108W/+*^ mice (Appendix C Supplementary Table 4); urinary corticosterone was increased (Appendix C Supplementary Table 5). Although we did not observe differences in *Crh* expression, OTP has been reported to be involved in stress-induced regulation of *Crh* [26].

### 3.6 *Otp*^*R108W/+*^ mice exhibit anxious behavior

As oxytocin and vasopressin can modulate social behaviors (see for example [27], [28]), we conducted a number of behavioral tests in *Otp*^*R108W/+*^ mice and wild type littermates that were singly housed or group housed. In an open field test, *Otp*^*R108W/+*^ mice spent significantly less time in the center of the arena than wildtype littermates (Figure 4E). Similarly, in the light–dark box test, *Otp*^*R108W/+*^ mice spent significantly less time in the light zone compared to wild type littermates (Figure 4F). These data are consistent with increased anxiety in *Otp*^*R108W/+*^ animals, which may in part explain increased urinary corticosterone levels. *Otp*^*R108W/+*^ mice showed significantly reduced locomotor activity in the open field and light–dark box whereas there were no differences in activity between genotypes in group housed animals (Figure 4G), suggesting that the anxiety phenotype in these animals can be precipitated by novel environments or social isolation.

### 3.7 Rare missense variants identified in severely obese humans

**Figure 5:**
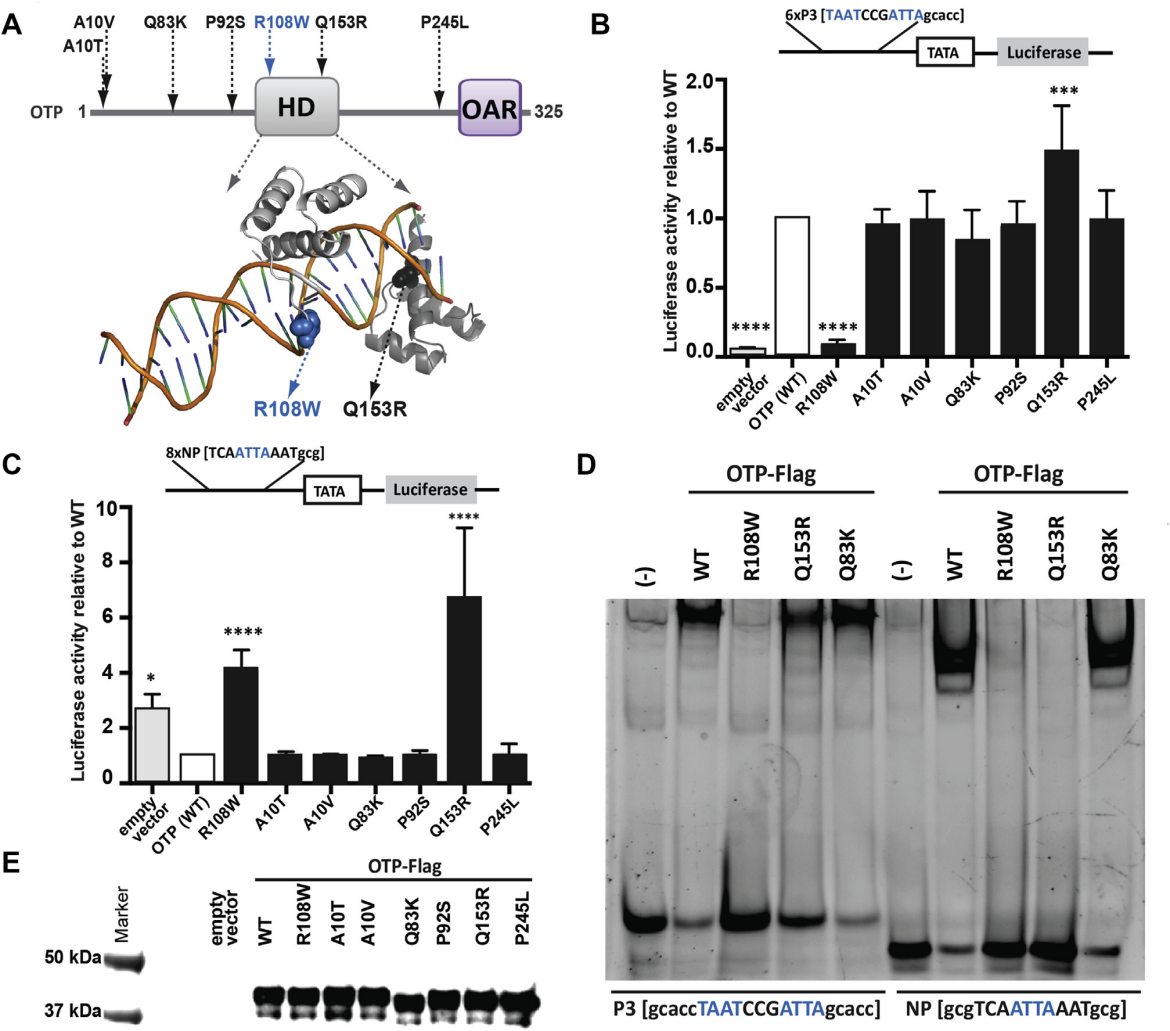
**Functional characterization of OTP variants**. (**A**) Schematic representation of the human OTP protein depicting the characteristic homeobox (HD) and OAR (otp, aristaless, and rax) domains. Human variants (black) and the mouse variant (blue) identified in this study are shown. (**B**) Homodimeric human OTP binding tested using a palindromic TAAT 6xP3 reporter [30]. (**C**) Monomeric OTP binding tested with a 8xnp reporter [10]. Reporter schematics and response elements are shown. Data are the average of 6 independent experiments; luciferase activity was normalized to WT OTP. Mean ± standard deviation are shown; statistical significance tested by ANOVA; *p < 0.05; ***p < 0.001, ****p < 0.001. (**D**) Effect of OTP variants on DNA binding to palindromic P3 and single site NP DNA probes. Nuclear extracts from HEK293-T cells transiently transfected with WT/mutant OTP variants incubated with FAM labeled DNA. (−) represents non-transfected control. (**E**) Expression of Flag-tagged WT/mutant OTP in transiently transfected HEK293-T cells. Compared to WT, the Q83K mutant consistently showed different electrophoretic mobility.

We next investigated the potential role of OTP in humans by analyzing exome sequencing and targeted resequencing data on 2548 European ancestry individuals with severe, early-onset obesity recruited to the Genetics Of Obesity Study (GOOS; mean body mass index [BMI] standard deviation score 3; age of onset <10 years) and 1,117 ancestry-matched controls (Methods) [21]. We identified 4 rare heterozygous missense variants; no additional rare variants were found uniquely in the control dataset. The most frequent variant in our sample (Q83K; rs143794465) was present in ExAC with an allele frequency of 0.28% across all ancestries (0.46% in European ancestry). Burden association tests of the four variants in *OTP* and of Q83K did not reveal a significant association with obesity in our study (p-value = 0.15 and 0.35 respectively) (Appendix C Supplementary Table S6). Further inspection of *OTP* in an additional 935 GOOS cases identified 4 additional rare heterozygous missense variants, altogether detecting 8 variants in OTP in our severe obesity cohort. Five of these variants have not been reported in the ExAC database (http://exac.broadinstitute.org) [29] (Figure 5A). Interestingly, in ExAC, OTP is predicted to be highly intolerant to loss of function variation [29].

### 3.8 Functional characterization of human and mouse mutations in OTP

We tested the functional consequences of the novel human *OTP* mutations and Q83K as well as the mouse R108W mutant; all affect residues that are highly conserved across species (Appendix B Supplementary Figure 3a and b). First, we performed docking studies of the OTP homology model, based on human Brn-5 transcription factor in complex with *CRH* gene promoter (PDB code 3d1n). These showed similar interactions with DNA bases, namely Q153 and N154 in the major groove and, R106 and R108 in the minor groove (Appendix B Supplementary Figure 3c–e). The flexible linker region between the N-terminal POU-like domain (containing residues Q153 and N154) and the DNA-binding domain (containing residues R106 and R108) appears to be an essential structural feature to allow for multiple binding modes of these domains with DNA. In cells, none of the variants affected the nuclear localization of OTP (Appendix B Supplementary Figure 4). As gene targets of OTP are largely unknown [26], we used luciferase reporter assays based on consensus sequences for binding of Drosophila *Otp* [10] (Figure 5B and C). Two variants (mouse R108W and human Q153R) differed from WT in reporter assays, R108W completely abolishing and Q153R enhancing activity upon homodimeric binding to a palindromic response element (Figure 5B). On a single site response element, wild-type OTP was found to act as a repressor; both R108W and Q153R abolished this repression (Figure 5C). Both variants affect residues in the DNA binding homeodomain; in electrophoretic mobility shift assays (EMSAs), R108W abolished DNA binding, whilst Q153R maintained binding to the palindrome but lost binding to the single recognition site (Figure 5D**)**. The rare variant Q83K did not affect DNA binding (Figure 5D) but consistently ran slightly faster than WT on SDS-PAGE gels (Figure 5E), an effect that could be explained by altered post-translational modification. The other OTP variants did not differ from WT in the assays used but may affect function on endogenous promoters, particularly as our studies reveal that OTP can function as either a transcriptional activator or repressor dependent on target DNA sequence. We found no evidence for potential dominant negative effects via altered dimerization with WT OTP (Appendix B Supplementary Figure 3f and g).

## 4 Discussion

In a series of studies, we have demonstrated that the transcription factor OTP plays a critical role in the development of hypothalamic circuits involved in energy homeostasis. Given the small number of variants identified and the limited clinical information we have on OTP variant carriers (Appendix C Supplementary Table 7), additional replication will be required to investigate the potential contribution of these variants to obesity. This can be challenging when studying very rare/private alleles. Of note, heterozygous loss of function mutations in the transcription factor SIM1, which plays a very similar and overlapping role in the development of neurons in the PVN of the hypothalamus, are more prevalent and associated with hyperphagia, severe obesity and behavioral abnormalities [2], [3], [5], [6]. Further characterization of the transcriptional network that regulates hypothalamic development and the identification of downstream target genes may shed light on the role of these specific neuronal populations in energy homeostasis and behavior.
