# Supplementary material for: Disruption of the homeodomain transcription factor orthopedia homeobox (Otp) is associated with obesity and anxiety
Source: Mol Metab. 2017 Aug 24;6(11):1419–28. doi: 10.1016/j.molmet.2017.08.006 (PMC5681237; doi:10.1016/j.molmet.2017.08.006)
Supplement: Supplementary file 1 [file mmc1.docx]

**Appendix A**

Disruption of the homeodomain transcription factor orthopedia homeobox (*Otp*) is associated with obesity and anxiety

Moir *et al.*

**Supplementary Materials and Methods**

**Mouse studies**

**Strains and husbandry**

All animal studies were performed under the guidance issued by the Medical Research Council (MRC) in Responsibility in the Use of Animals for Medical Research (July 1993) and Home Office Project Licences 30/3146 and 30/3384. Mice were kept under controlled light (light 7am–7pm, dark 7pm–7am), temperature (21±2°C) and humidity (55±10%) conditions. They had free access to water (9–13 ppm chlorine) and were fed ad libitum on a commercial diet (SDS Rat and Mouse No. 3 Breeding diet, RM3) containing 11.5 kcal% fat, 23.93 kcal% protein and 61.57 kcal% carbohydrate. Following identification of the *Otp* mutation, mouse cohorts were maintained on a C3H/HeH background (C3H/HeH-OtpR108W/H). *Otp^R108W/+^* were crossed to C57BL/6J and maintained by backcross to generate mice for complementation tests. *Otp^tm1Asim/Cnrm^* mice were generated as previously described [1], imported through the European Mouse Mutant Archive (EMMA) and derived onto a C3H/HeH and C57BL/6J background. *Otp^tm1Asim^* mice phenotyping studies were performed on animals backcrossed to C3H/HeH 6 or 7 times, therefore >99 % of the genetic background comprised of C3H/HeH DNA. *Otp^tm1Asim^* mice maintained by backcross on a C57BL/6J background were used for complementation studies.

**Experimental Design**

Cohorts were bred for separate dedicated phenotyping experiments. Cohort sizes were estimated using power calculations on data from tests applied to other genetic mouse models of impaired glucose tolerance. A cohort of 13 *Otp^+/+^* and 9 *Otp^R108W/+^* males and 17 *Otp^+/+^* and 17 *Otp^R108W/+^* females congenic on a C3H/HeH background were bred for body mass and composition, glucose, insulin and leptin studies. A separate cohort of 8 *Otp^+/+^* and 7 *Otp^R108W/+^* males and 9 *Otp^+/+^* and 11 *Otp^R108W/+^* females was bred to investigate insulin sensitivity. To comprehensively investigate energy balance a cohort of 10 *Otp^+/+^* and 10 *Otp^R108W/+^* males were bred. A separate cohort was bred for behavioural phenotyping. 9 *Otp^+/+^* and 10 *Otp^R108W/+^* males congenic on a C3H/HeH background were bred for open field, light/Dark box. In *Otp^tm1Asim^* mice a cohort of 10 *Otp^+/+^* and 12 *Otp^+/tm1Asim^* males and 10 *Otp^+/+^* and 10 *Otp^+/tm1Asim^* females were bred for body mass and composition, glucose, insulin and leptin studies. A separate cohort of 7 *Otp^+/+^* and 10 *Otp^+/tm1Asim^* males and 6 *Otp^+/+^* and 10 *Otp^+/tm1Asim^* females were bred to investigate insulin sensitivity. For complementation testing, several *Otp^R108W/+^* males and *^Otp+/tm1Asim^* female mice on a C57BL/6J background were mated to confirm *Otp^R108W/tm1Asim^* mice at birth (P0/P1) and at weaning (P21). Animals were not randomised and the operators were not blinded.

**Genotyping**

Genome mapping was carried out using a KBioscience panel of 91 SNP’s that were informative between C57BL/6J and C3H/HeH.

*Otp^R108W^* mice were genotyped for the mutation by pyrosequencing using a PSQ HS96 machine (Qiagen) and run according to the manufacturer’s instructions. Primers: Forward 5’Biotin- GTCGAAGGAGCCACTCTG-3’, Reverse 5’- AGATGTCGGGGTAGTGAGTC-3’, Sequencing 5’- TGGGGTGAAGCGTGT-3’. *Otp^tm1Asim/Cnrm^* mice were genotyped using Taqman probe copy count qPCR. The qPCR assays for wild-type or LacZ (marking the knockout allele), were FAM labelled and run in duplex on a TaqMan system based on real-time detection of accumulated fluorescence (ABI Prism 7900, Applied Biosystems) with a VIC labelled internal control dot1l. Results were analysed and copy counted using the ABI CopyCaller software v.2. (ABI). Ct values of greater than 30 were called as a fail. Each sample was run with a technical duplicate and each assay run with 7 controls of known genotype and a blank control.

**Body Mass and Composition**

Body mass was measured weekly using scales calibrated to 0.01 g. Analysis of body composition was performed using an Echo MRI whole body composition analyser (Echo Medical System, Houston, TX) every 2 weeks from 4 weeks of age. Dual-energy X-ray Absorptiometry (DEXA, PIXImus, Wisconsin, USA) was used to quantify fat mass, lean mass and bone mineral content and density on euthanised animals.

**Fasted bleeds**

After a 5 hour fast blood was sampled from the lateral tail vein under local anaesthetic. Plasma insulin and leptin was assayed using a Mouse MILLIPLEX kit (Millipore).

**Intraperitoneal glucose tolerance test (IPGTT)**

IPGTT’s were carried out according to the IMPReSS protocols (https://www.mousephenotype.org/impress/protocol/87/8) using 2 g glucose per kg body weight. Mice were fasted overnight and blood sampled under a local anaesthetic at 0 minutes (baseline) and 60 and 120 minutes post glucose injection. Plasma blood glucose was measured using a GM9 glucose analyser (Analox Instruments, London, UK). Plasma insulin and leptin was assayed using a Mouse MILLIPLEX kit (Millipore).

**Intraperitoneal insulin tolerance test (ipITT)**

Animals were fasted for 5-6 hours and blood sampled under a local anaesthetic at 0 minutes (baseline), prior to injection of 1.5 IU insulin per kg of body weight, and subsequently at 15, 30, 45, 60 and 90 min. Whole blood glucose was measured using an AlphaTRAK meter and test strips (Abbott Animal Health).

**Free fed bleeds**

The test was undertaken at the same time of day (afternoon). For plasma oxytocin measurements blood was sampled from the lateral tail vein into Lithium-Heparin microvette tubes (Sarstedt) under local anaesthetic and assayed using a Mouse ELISA kit (Cusabio Biotech, Stratech). Measurement of the thyroid hormones was carried out on serum collected by cardiac puncture after the mice had been given a lethal dose of anaesthetic, sodium pentobarbitone (Pentoject, Animalcare, York, UK). Once fully anaesthetised a 22G needle was inserted through the chest wall into the heart and blood drawn up into a 1 ml syringe. The needle was removed and the blood drawn from the syringe, by capillary action, into EDTA microvette tubes (Sarstedt). The following ELISA’s were used following manufacturers instructions; T3 (Total) Mouse/Rat ELISA kit (Abnova, Taiwan), Mouse/Rat T4, Total ELISA (Calbiotech Inc, USA) .

**Urine Collection**

Spot urine was collected into a petri dish and then transferred to a 1.5 ml tube in the hour before lights out (between 6 and 7 pm) by the same person to reduce stress. Urinary concentrations of corticosterone were quantified using an AssayMax ELISA kit (AssayPro, USA).

**Energy Balance Studies**

To measure food intake male mice of each genotype; wild-type and heterozygote were housed into pairs of the same sex and genotype from 4 weeks of age until experiment concluded [2]. Each cage was given a weighed (approximately 80 g) amount of diet. Diet was reweighed daily (Monday – Friday) between 9 and 10 am on a balance which was accurate to 3 decimal places (Ohaus Explorer® Pro, Ohaus Europe GmbH, Switzerland), checking for spillage. The amount of food was topped up with fresh diet to approximately 80 g and returned to the mice. Division of the amount eaten by two gave a per mouse amount of food consumed in 24 hours. A calculated intake was determined for Saturday and Sunday from the food remaining on Monday morning.

Once a week on the same day the mice were put into a fresh cage and 24 hours later into a further fresh cage. The faecal samples from the 24 hours were collected from each cage. Faecal samples were dried at 55 °C for 48 hours. Approximately 1 g (weighed accurately to 3 decimal places) was burnt in a bomb calorimeter (IKA® C2000 Basic Calorimeter, Staufen, Germany) for determination of energy content of the faeces. Using the known weight of the dry sample the 24 hour faecal energy output was calculated as follows:

Faecal Energy kJ/g = Dry Sample Weight (g) x Energy of Sample (kJ)

To calculate energy intake, the gross energy of the diet was determined as 15.0 kJ/g from burning 1 gram of dried RM3 pellets on three separate occasions in the bomb calorimeter and the food intake for the 24 hour period corresponding to the faecal collections was used. Food and faecal weights were per cage values; the value was divided by two to establish a ‘per mouse’ value for use in further calculations.

Energy Intake (EI) ((kJ/g)/day) = Food Intake (g/day) x Energy Food Intake (kJ⁄g)

Further calculations were carried out using the following equations:

Digested Energy Intake (DEI) (kJ/g) = EI - Faecal Energy

Metabolisable Energy Intake (MEI) (kJ/g) = DEI -2% (lost as energy in urine*)

Apparent Energy Assimilation Efficiency (%) = (DEI/EI ) x 100 %

*Energy loss through urine was not measured and was assumed to be equal between groups and small (2 %) [3].

Energy Balance Calculations: EE was extrapolated to produce values for 24 hours (light adjusted EE values were multiplied by 12 and added to dark adjusted EE values also multiplied by 12). The daily EE value was subtracted from its corresponding MEI value for its age to give a surplus energy value. Multiplication by 7 gave energy surplus values over a week. Subtraction of mean wild-type values for the week from heterozygote values provided differences between the two that indicated if heterozygotes were in positive or negative energy balance that week.

Protocols for energy balance studies are described in more detail by Moir et al. [2]

**Indirect Calorimetry**

Metabolic rate was measured once a week between 4 and 16 weeks of age using indirect calorimetry (Oxymax, Columbus Instruments)to determine oxygen consumption, carbon dioxide production, respiratory exchange ratio (RER) and heat production. Heat production (energy expenditure) was calculated using; Heat = CV x VO2, where CV = 3.815 + 1.232 x RER (CV, calorific value based on the observed RER; Oxymax, Columbus Instruments). Adjustment was made for variation in lean mass using multiple linear regression analysis (ANCOVA). The following phenotypes were adjusted; VO2, VCO2, and Energy Expenditure (EE). The linear model used is outlined in McMurray *et. al.,* [4].

**Behavioural phenotyping**

Open field behaviour: Full details of the open field analysis are given on the Impress database (https://www.mousephenotype.org/impress). Briefly, individual mice were placed into one corner of a walled arena (grey polyvinyl chloride; 45cm X 45cm), facing the side wall. Animals were allowed to explore the arena for 20 minutes. During this time animal movements were tracked by digital camera and analysed automatically by EthoVision XT analysis software (Noldus).

Light/Dark box: Individual mice were placed into one corner of an enclosed arena (overall dimensions 40cm X 40cm). One half of the arena (light side) is open to the light with walls made from transparent polyvinyl chloride (dimensions 20cm X 40cm). The other half of the arena (dark side) is fully enclosed with a lid to prevent illumination with the walls and lid made from black polyvinyl chloride (dimensions 20cm X 40cm). The light and dark sides are connected by a small opening (4cm X 4cm) in the centre of the separating wall. Animals were allowed to explore the arena for 20 minutes. Animal movements and position were monitored by EthoVision XT analysis software (Noldus).

Home cage activity monitoring: Group housed animals were monitored as described in [5]. Briefly group housed mice (three per cage) were tagged with RFID micochips and placed in a home cage analysis system which captured movement of mice by both video tracking and tracking of RFID chips. Individually housed animals were monitored as previously described in [6]. Briefly, mice were singly housed and placed in light controlled chambers with near-infrared (NIR) miniature CCD cameras positioned above the cages (Maplin, UK). Monitoring during dark periods was performed using infrared illumination. Video monitoring was performed for a 24 hour period over a 12 hour light/dark cycle (100 lux light intensity). Video files were uploaded to ANYmaze video analysis software (Stoetling) for activity analysis.

**Quantitative RT-PCR**

RNA was extracted from snap frozen tissues collected between 9 and 10am from free fed mice using an RNeasy Mini Kit (Qiagen, UK) according to the manufacturer’s instructions. cDNA was generated using Superscript II enzyme (Invitrogen, UK) and analysed by quantitative RT-PCR using the TaqMan system based on real-time detection of accumulated fluorescence (ABI Prism 7700, Perkin-Elmer Inc., USA). To select the appropriate house-keeping genes geNorm analysis using 12 reference genes (Primerdesign UK) was undertaken against all samples. Samples were tested in triplicate and gene expression normalised relative to the expression of house-keeping genes *Sdha* and *Ubc* for 4 weeks, *Ubc* and *Ywhaz* for 8 weeks and *Rpl13a* and *Sdha* for 10 weeks. Fam labeled Taqman Probes were purchased from Life Technologies (ABI, USA): Otp (Mm00440574_m1) Agrp (Mm 00475829_g1), Avp (Mm01271704_m1), Oxt (Mm01329577_g1), Pomc (Mm00435874_m1), Rpl13a (Mm01612986_gH), Sdha (Mm01352366_m1), Sst (Mm00436671_m1), Trh (Mm01182424_mH), Ubc (Mm01201237_m1), Sim1: Mm00441390_m1, Ghrh: Mm00439100_m1 and Ywhaz (Mm01722325_m1).

**Western Blotting**

Total protein was extracted from the hypothalamus of P0 *Otp^+/+^*, *Otp^R108W/+^*, *Otp^+/tm1Asim^* and *Otp^R108W/tm1Asim^* mice. Briefly, hypothalamic tissue was placed in lysis buffer (CelLytic MT Cell lysis Reagent, Sigma) containing phosphatase (Roche) and protease (Roche) inhibitor cocktails. Samples were homogenised with a pellet pestle mortar (Kimble Chase, New Jersey, USA) and centrifuged at 10 000 x g at 4 °C for 45 minutes. The supernatant was removed and protein concentrations were estimated using the Bio-Rad Dc Protein assay.

For western blot analysis, 10 μg of the protein lysates were run on 12% Bis-Tris SDS–PAGE gels (NuPAGE Novex, Invitrogen) and transferred to nitrocellulose membranes. Membranes were blocked for 1 h at room temperature in 5% milk in TBS-T, and incubated overnight at 4 °C with primary antibodies to Rabbit anti-OTP antibody at 1:750 (HPA039365, Sigma Prestige Antibodies ®), Mouse 12G10 anti-alpha-tubulin 1:15 000 (Developmental Studies Hybridoma Bank, University of Iowa, USA). Blots were washed in TBS-T and incubated with secondary antibody at 1:3000 (horseradish peroxidase (HRP)-anti-rabbit IgG for OTP, HRP-anti-mouse IgG for alpha-tubulin, Bio-Rad) in 5% milk in TBS-T for 1 hour at room temperature. After washing, bands were visualized with Thermo Fisher Scientific Pierce™ ECL 2 Western Blotting Substrate (Thermo Fisher Scientific) and X-Ray film. Protein density was analysed using the Image Lab software (version 5.1, Bio-Rad).

**Tissue Processing**

For immunohistochemical analysis, adult mice (10 weeks old) were sacrificed by cervical dislocation and the brain was dissected and immediately immersed in cold 4 % paraformaldehyde (PFA)-PBS, incubated at 4 °C for 24 h, and then in 30 % sucrose-PBS at 4°C until sinking (typically 2-3 days). Brains were then blotted to remove moisture and frozen on dry ice before storage at -80 °C. Coronal cryosections were cut at 10 µm and collected on charged slides. Slides were allowed to air dry before storage at -80 °C.

Day 0 mice were necropsied and fixed in normal buffered saline. Samples of major organs were prepared to make H&E stained 5 µm sections which were then examined by a pathologist.

**Immunohistochemistry**

The immunoreaction of OTP, OXT and AVP were detected by immunohistochemical techniques. Briefly slides were thawed on ice and fixed in 4 % PFA-PBS for 10 minutes. Following washes in PBS, slides were briefly washed in dH_2_O, and antigen retrieval was carried out in 10mM Tris, 1mM EDTA, pH 8 and microwaved on full power for 10 minutes. After allowing to cool at room temperature, slides were again washed in PBS and permeablised for 10 minutes with 0.2 % Triton X-100 (Sigma) in PBS. Following permeablisation, slides were washed in PBS and blocked with 10 % serum-PBS, 0.5% Tween-20 (PBST) for 1 h at room temperature, using serum corresponding to the species secondary antibody was raised in (AVP, OTP: donkey serum, OXT: goat serum; Sigma). For co-localisation experiments, a combination of 5% serum from each species were used. Slides were then incubated at 4 °C with either Rabbit Anti-OTP (1:250, HPA039365, Sigma Prestige Antibodies ®), Guinea Pig Anti-OXT (1: 500, PA1-18416 Thermo Fisher Scientific), or Rabbit Anti-AVP (1:1000, AB1565, Millipore) primary antibody diluted in blocking serum. For co-localisation experiments, slides were incubated with Anti-OXT and Anti-OTP together in the same concentrations as above. The following day, slides were washed three times in PBST and re-blocked for 1 h at room temperature. Sections were then incubated in the dark for 1 h with appropriate fluorescent secondary antibody (Alexa Fluor® 488 Donkey Anti-Rabbit IgG (H+L), A-21206 at 1: 250, Life Technologies, Alexa Fluor® 568 Goat Anti-Guinea Pig IgG (H+L), A-11075 at 1: 250, Life Technologies), or in combination for co-localisation experiments, in blocking serum. Slides were washed in PBST in the dark, before a final wash in PBS and incubated with with NucBlue Stain (R37606, Invitrogen), washed with PBS, and mounted with a fluorescence mounting media (DAKO) and coverslipped. Slides were then stored at 4 °C in the dark until imaging not more than 1 week later.

**Imaging and PVN Cell Counts**

Sections were visualised by confocal imaging using an LSM 700 inverted microscope (Zeiss), which is equipped with four lasers; blue near UV diode 405 nm, blue 488 nm, green 561 nm and red 633 nm and Zen 2012 (black) imaging software. Cell counts were determined using ImageJ software (<http://imagej.nih.gov/ij/>). Coronal sections corresponding approximately to bregma -0.94 mm, bregma -0.82 mm, and bregma -0.70mm [7] were imaged and left and right PVN were counted separately, and thus 6 counts were averaged for each animal for each immunostain. For co-localsation experiments, OXT neurons co-localising with OTP expressing neurons were expressed as a percentage of OXT positive neurons for comparison.

**Statistics**

**Outliers:** In Figure 1e and S1a, male and female bodyweight, there was in each case one outlier heterozygous mouse losing or failing to gain weight from 12 and 9 weeks respectively, however in each case it was not removed as this likely reflects a real biological effect resulting from a loss of condition due to diabetes. In figure 1i IPGTT the male heterozygote data contains one outlier mouse with much lower glucose in the challenge by visual inspection of the scatter of values, we verified the genotype of this mouse and so it may reflect a technical problem and was therefore excluded, however the difference between groups is highly significant regardless of the inclusion or exclusion of that individual.

**Data inspection:** *In vivo* phenotyping data was visualised in Graphpad Prism 6.0g and assessed for normal distribution using a D’Agostino and Pearson omnibus normality test and for similar variance using the Brown-Forsythe test.

**Statistics:** Statistical tests were selected depending on how well data sets conformed to normality and equal variance and are indicated in the figure legends and were carried out using Graphpad Prism 6.0g. Any data transformations are also indicated in the legends. The tests used included the following: 2-way repeated measure ANOVA with Bonferroni post hoc tests for comparison of genotypes for longitudinal data that showed equal variances. Unpaired two-tailed *t*-tests with Welch's correction for unequal variance were used to investigate the difference between the means of two genotype classes and paired t-tests for between time-point comparisons. Similarly, non-parametric unpaired Mann-Whitney 2-tailed or Wilcoxon matched–pairs signed rank tests were used for data that were not normally distributed. In some cases Area Under the Curve (AUC) was calculated, baselined to each first measurement except in the case of ipITT tests where the baseline was zero, for longitudinal data to allow simple pairwise comparisons using non-parametric tests. Chi-squared tests were used to compare the difference between the observed and the expected number of mice. Indirect calorimetry data was adjusted for variation in lean mass and analysed using multiple linear regression analysis (ANCOVA), which was performed using R statistical software (R version 2.13.1).

**Data availability:** Mouse phenotyping data available on request.

**Human Studies**

**Ethical approval and data sets**

Ethical approval for studies was given by the Cambridge Research Ethics Committee; all participants gave written informed consent. The Genetics of Obesity Study (GOOS) is a cohort of 7,000 individuals with severe early-onset obesity; age of obesity onset is less than 10 years. Severe obesity is defined as a body mass index (weight in kilograms divided by the square of the height in meters) standard deviation score greater than 3 (standard deviation scores calculated according to the United Kingdom reference population). Targeted Sequencing (TS) and Whole Exome Sequencing (WES) was performed as described as part of the UK10K consortium [8]. Data was compared to subsets within the neurodevelopmental and rare disease groups that were consented for use as controls. Details and further information about the UK10K project can be found at <http://www.uk10k.org/> and in the UK10K consortium paper from 2015 [8]. Variants found through whole exome sequencing were confirmed in genomic DNA of probands and subsequently family members by Sanger sequencing (primers and conditions available on request).

All variant annotation was applied using the GRCh37 human reference. Variants were annotated with rsIDs from dbSNP 137, and allele frequencies from the final 1000Genomes Phase 1 integrated (v3) callset, the NHLBI Exome Sequencing Project (ESP) v2, and the UK10K WGS sample set. The Ensembl Variant Effect Predictor v2.8 with Ensembl 66 was used to add variant consequence annotations including the predicted deleteriousness of each missense variant as predicted by SIFT, PolyPhen, and Condel. To identify ***rare*** variants, we used the thirteen UK10K WES sample sets that were not obese sample sets and were not included as controls in this study. We removed variants that had a MAF > 1% across all or a MAF > 10% in any of the thirteen sample sets. We then removed variants with a MAF > 1% in any of seven additional sample sets: 1. UK10K WGS sample set, 2-3. European and African American NHLBI ESP v2 sample sets, and 4-7. the four continent sample sets (AFR, AMR, ASN, EUR) from 1000 Genomes Phase 1 integrated v3 callset. ***Novel*** variants were identified as sites not seen in any of the internal or external datasets used for MAF annotation. We implemented the optimal Sequence Kernel Association Test SKAT-O [9, 10] with the SKAT R package v1.1.2 [11] using options *method=”optimal.adj”* for SKAT-O and *r.corr=1* for burden.

**Structural Analysis of OTP Variants**

The structural representations using ribbon-depicted models were generated using the Open-Source PyMOL Molecular Graphics System, Version 1.7.x Schrödinger, LLC (http://pymol.org).

In **Supplementary Figure** **3b**, A Blastp search, based on the full length OTP sequence, revealed 56 reviewed OTP homologs in the UniprotKB sequence database, from which a multiple sequence alignment of these 56 OTP homologs was generated and used to create the corresponding sequence logo (**Supplementary Figure 3b**) corresponding to residues 104-163 (<http://weblogo.berkeley.edu/logo.cgi>). In **Supplementary Figure 3** the protein fold recognition server, Phyre2[12] was used to generate an OTP homology model (residues 13-161). Molecular docking of OTP and DNA was performed using the information-driven, on-line DNA-docking server HADDOCK Ver. 2.2[13].

**Functional characterisation of human variants in OTP**

**Expression plasmids**

Human cDNA of human OTP with C-terminal 3XFlag tags and respective gene variants were synthesized and cloned into pcDNA5/Frt/TO (Invitrogen), and Luciferase reporter constructs 8xnp and 6xP3 were synthesised and cloned into vector pLuc-MCS (Stratagene) by GenScript (NJ, USA). Presence of the desired mutation and the fidelity of each construct were confirmed by Sanger sequencing.

**Luciferase gene reporter assays**

HEK293-T cells were plated in D-lysine coated 96-well cell culture plates at a density of 1 × 10^4^ cells/well, and grown in DMEM media (Sigma) supplemented with 10% foetal bovine serum, 2 mM L-glutamine and 1 mM Penicillin/Streptomycin (Sigma). After overnight culture, media was changed to serum free Opti-MEM and wells were transfected in triplicate with a 10:1 ratio of expression vector : reporter gene using Lipofectamine2000 reagent (Thermo Fisher Scientific) in Opti-MEM media, following manufacturer’s instructions. After 24 h, the cell medium was aspirated; cells washed with 150 uL Dulbecco’s PBS (with Ca2+ and Mg2+) (Sigma), then 50 uL Dulbecco’s PBS (with Ca2+ and Mg2+) + 50 uL Steadylite luciferase reagent (Perkin Elmer) were added to each well. Following 20 minutes incubation, luminescence was measured on a TopCount Microplate Scintillation and Luminescence Counter (Packard, UK) and readings of triplicate wells averaged to get values for empty vector, Wt OTP and each OTP variant.

**SDS PAGE and Immunoblot analysis**

Aliquots from the Luciferase reporter assay lysates (5 uL) were mixed with sample loading buffer, denatured at 95^o^C for 5 mins, separated by SDS-PAGE on 4-12% Novex Tris-Glycine gels (Life Technologies) at 120 V for 30 mins, and then transferred onto nitrocellulose membrane using the iBlot system (Life Technologies). Membranes were incubated for an hour in TBST buffer (1× TBS plus 0.1% Tween-20) with 5% dry milk at room temperature before addition of the primary antibody: monoclonal Flag M2 antibody (Sigma) diluted to 1:1000 in 1% dry milk and incubated for an hour. Membranes were washed 3 times in TBST buffer, and secondary antibody incubation was performed for an hour with horseradish peroxidase–conjugated anti-mouse IgG (Dako) diluted (1:2000) in TBST buffer/ 1% dry milk. Protein expression was visualized with SuperSignal ECL reagent assay (Thermo Fisher Scientific) and detection carried out using Chemidoc Digital Imager (Bio-Rad).

**Electrophoretic Mobility Shift Assays**

Wt or variant OTP-3xFlag expression vectors were transfected into HEK293-T cells (courtesy of Dario Alessi, Dundee and mycoplasma tested) and after 72 hr, harvested cells were resuspended in hypotonic buffer (10mM Hepes pH 8.0, 1.5mM MgCl_2_, 10mM KCl, 0.4% Igepal, 10% ficoll, 1x protease inhibitor cocktail, 1mM DTT), incubated on ice for 5 mins and then centrifuged at 14K rpm for 30 mins at 4 ^o^C. The cytosol was removed and nuclear pellets lysed with plunger tips in nuclear extract buffer (20mM Hepes pH 8.0, 1.5mM MgCl_2_, 420mM KCl, 20% glycerol, 0.2mM EDTA, 1x protease inhibitor cocktail, 1mM DTT), shaken for 30 min at 4 ^o^C and then centrifuged at 14K rpm for 30 mins at 4 ^o^C. Nuclear extracts (30 μg of protein) were incubated with 40nM 6-FAM 5’ labeled oligo (P3-gcaccTAATCCGATTAgcacc or NP-gcgTCAATTAAATgcg) and 0.1 μg/μl of poly[d(I-C)] in EMSA buffer (20mM Tris-HCl pH 8.0, 150mM NaCl, 3mM MgCl_2_, 8% glycerol, 5 mM dithiothreitol, final volume of 30 uL) for 30 mins at 25 ^o^C. DNA-protein complexes were analysed on a 7% polyacrylamide gel in 50 mM Tris-HCl, pH 8.4/380 mM glycine/2 mM EDTA, and imaged using Chemidoc Digital Imager (Bio-Rad).

**Cell Imaging**

Transfected HEK-293T cells plated on 35 mm, poly‐d‐lysine‐ coated glass cover slips were incubated for 24h in growth medium. Cells were fixed using 4% formaldehyde and permeabilised in 0.1% Triton X100, followed by immunostaining with monoclonal Flag M2 antibody (Sigma) diluted to 1:100, with secondary α-mouse Alexa Fluor 568 (Thermo Fisher Scientific) and DAPI (Thermo Fisher Scientific) stained. Cells were visualized with Confocal Laser Scanning Platform Leica TCS SP8 microscope (Leica Microsystems) using a 63x/1.40 NA Plan Apo oil immersion objective and LASX suite software (Leica Microsystems). Individual images were processed and analysed using ImageJ software (<http://imagej.nih.gov/ij/>).

**Statistical analysis**

Data from multiple biological replicate experiments was analysed (n=6). Statistical significance was established by ANOVA test with multiple comparison to the same reference sample wild-type (WT) OTP, and calculated in GraphPad Prism software. Statistically significant values were defined as P < 0.05.

1. Acampora, D., Postiglione, M.P., Avantaggiato, V., Di Bonito, M., Vaccarino, F.M., Michaud, J.*, et al.* 1999. Progressive impairment of developing neuroendocrine cell lineages in the hypothalamus of mice lacking the Orthopedia gene. Genes Dev 13(21):2787-2800.

2. Moir, L., Bentley, L., & Cox, R.D. 2016. Comprehensive Energy Balance Measurements in Mice. Curr Protoc Mouse Biol 6(3):211-222.

3. Drozdz, A. (1975) Food Habits and Food Assimilation in Mammals. *Methods For Ecological Bioenergetics*, eds Grodzinski W, Klekowski RZ, & Duncan A (Blackwell Scientific Publications, Oxford, United Kingdom).

4. McMurray, F., Church, C.D., Larder, R., Nicholson, G., Wells, S., Teboul, L.*, et al.* 2013. Adult onset global loss of the fto gene alters body composition and metabolism in the mouse. PLoS Genet 9(1):e1003166.

5. Bains, R.S., Cater, H.L., Sillito, R.R., Chartsias, A., Sneddon, D., Concas, D.*, et al.* 2016. Analysis of Individual Mouse Activity in Group Housed Animals of Different Inbred Strains Using a Novel Automated Home Cage Analysis System. Front Behav Neurosci 10.

6. Banks, G., Heise, I., Starbuck, B., Osborne, T., Wisby, L., Potter, P.*, et al.* 2015. Genetic background influences age-related decline in visual and nonvisual retinal responses, circadian rhythms, and sleep. Neurobiol Aging 36(1):380-393.

7. Franklin, K., B., J. & Paxinos, G. (2008) *The Mouse Brain in Stereotaxic Coordinates, Compact, 3rd Edition* (Elsevier Academic Press) 09 Apr 2008 Ed.

8. Consortium, U.K., Walter, K., Min, J.L., Huang, J., Crooks, L., Memari, Y.*, et al.* 2015. The UK10K project identifies rare variants in health and disease. Nature 526(7571):82-90.

9. Lee, S., Wu, M.C., & Lin, X. 2012. Optimal tests for rare variant effects in sequencing association studies. Biostatistics 13(4):762-775.

10. Wu, M.C., Lee, S., Cai, T., Li, Y., Boehnke, M., & Lin, X. 2011. Rare-variant association testing for sequencing data with the sequence kernel association test. Am J Hum Genet 89(1):82-93.

11. Anonymous 2015. Seunggeun Lee, with contributions from Larisa Miropolsky and Michael Wu. SKAT: SNP-Set

(Sequence) Kernel Association Test. . R package version 1.1.2. [http://cran.r-project.org/package=SKAT](http://CRAN.R-project.org/package=SKAT).

12. Kelley, L.A., Mezulis, S., Yates, C.M., Wass, M.N., & Sternberg, M.J. 2015. The Phyre2 web portal for protein modeling, prediction and analysis. Nat Protoc 10(6):845-858.

13. van Zundert, G.C., Rodrigues, J.P., Trellet, M., Schmitz, C., Kastritis, P.L., Karaca, E.*, et al.* 2016. The HADDOCK2.2 Web Server: User-Friendly Integrative Modeling of Biomolecular Complexes. J Mol Biol 428(4):720-725.
