# Supplementary material for: Disruption of the homeodomain transcription factor orthopedia homeobox (Otp) is associated with obesity and anxiety
Source: Mol Metab. 2017 Aug 24;6(11):1419–28. doi: 10.1016/j.molmet.2017.08.006 (PMC5681237; doi:10.1016/j.molmet.2017.08.006)
Supplement: Supplementary file 2 [file mmc2.docx]

**Appendix B**

Disruption of the homeodomain transcription factor orthopedia homeobox (*Otp*) is associated with obesity and anxiety

Moir *et al.*

**Supplementary Figures**


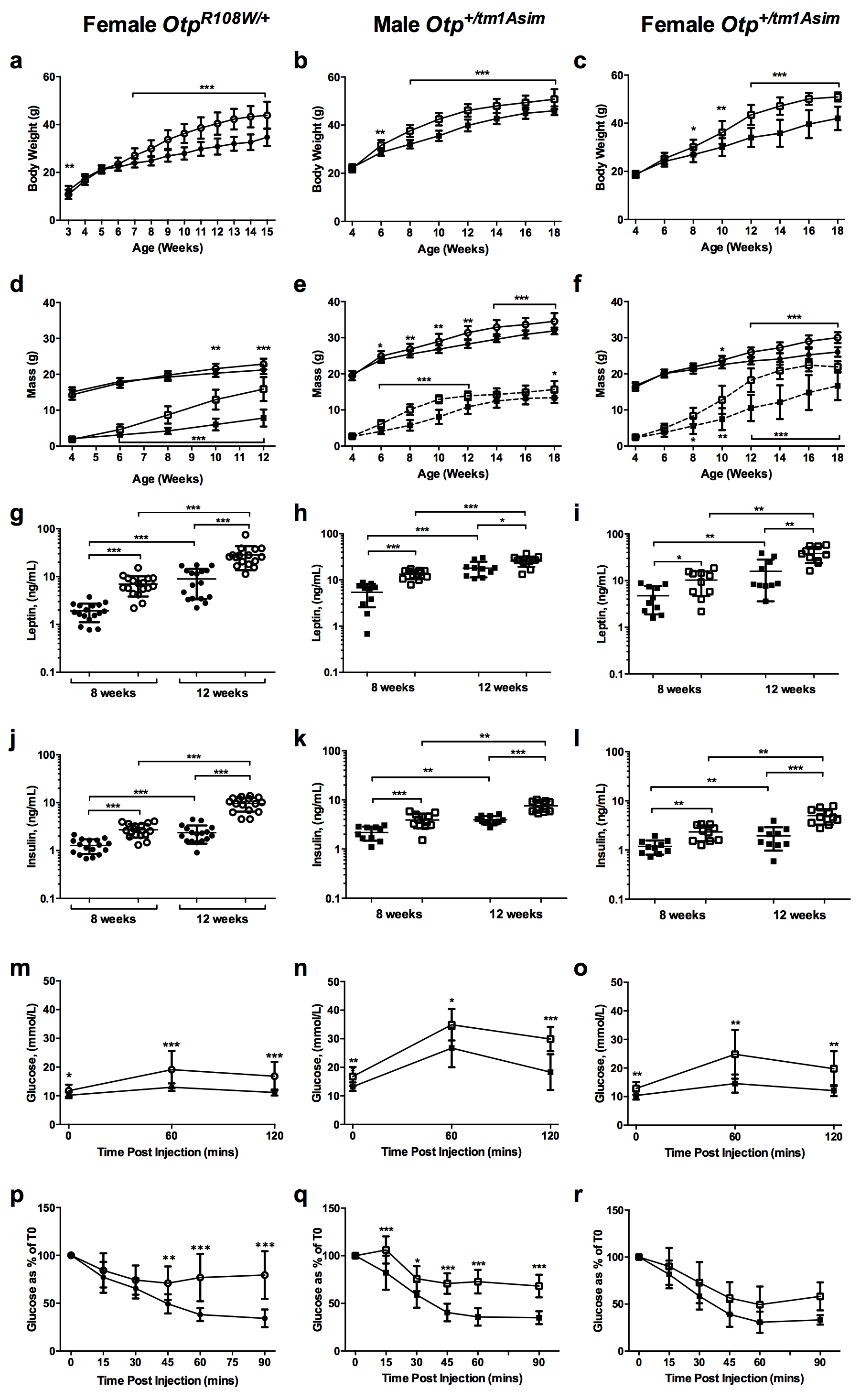


**Supplementary Figure 1. Related to Figure 2 a-f. *Otp^R108W/+^* mice are obese, glucose intolerant and insulin resistant and heterozygous knockout *Otp^+/tm1Asim^* mice on a C3H/HeH background exhibit a similar but milder phenotype.**

(**a**) Female *Otp^R108W/+^* weekly bodyweights showed significantly increased bodyweight from 7 weeks and evidence of lower average weight at 3 weeks. (**b,c**) Male and female fortnightly bodyweights of *Otp^+/tm1Asim^* mice showed significantly heavier bodyweight from 6 and 8 weeks of age respectively. (**d**) Weekly EchoMRI measurement of female *Otp^R108W/+^* mice showed significantly increased lean mass (circle) at 10 and 12 weeks of age and fat mass (square) from 6 weeks of age. (**e,f**) Male and female *Otp^+/tm1Asim^* fortnightly EchoMRI measurement, showed significantly increased lean mass (circle) from 6 weeks of age and increased fat mass (square) between 6 and 12 weeks and at 18 weeks of age in males and significantly increased lean mass from 10 weeks and fat mass from 8 weeks of age in females. (**g**) Female *Otp^R108W/+^* plasma leptin concentrations after a 4-6 hour fast at 8 and 12 weeks of age in mice were increased. **(h,i**) Male and female *Otp^+/tm1Asim^* plasma leptin concentrations after a 5-6 hour fast at 8 and 12 weeks of age were significantly increased in mice of both sexes. (**j**) Female *Otp^R108W/+^* plasma insulin concentrations after a 4-6 hour fast at 8 and 12 weeks of age were increased. (**k,l**) Male and female *Otp^+/tm1Asim^* plasma insulin concentrations after a 5-6 hour fast at 8 and 12 weeks of age were significantly increased in mice of both sexes. (**m**) Female *Otp^R108W/+^* IPGTTs after a 5-6 hour fast at 12 weeks of age showed significantly impaired glucose tolerance at all timepoints post glucose injection. (**n,o**) Male and female *Otp^+/tm1Asim^* IPGTT in mice respectively after a 5-6 hour fast at 12 weeks of age showed significant impaired glucose tolerance at all timepoints post glucose injection in both sexes. (**p**) Female *Otp^R108W/+^* ipITT was performed in the afternoon after a 5-6 hour fast at 12 weeks of age showed insulin resistance at all time points from 45 mins. (**q,r**) Male and female *Otp^+/tm1Asim^* ipITT was performed in the afternoon after a 5-6 hour fast at 12 weeks of age and male mice were insulin resistant at all time points from 15 mins and female*s* did not display significant insulin resistance.

**Key**

*Otp^R108W/+^* are open symbols and *Otp^+/+^* are filled symbols.

*Otp^+/tm1Asim^* are open symbols and *Otp^+/+^* are filled symbols.

Mean + SD. *P<0.05, ** P<0.01, *** P<0.001.

**Animal numbers**

(**a**) *Otp^R108W/+^* (n = 17) and *Otp^+/+^* (n = 17), (**b**) *Otp^+/tm1Asim^* (n = 12) and *Otp^+/+^* (n = 10) , (**c**) female *Otp^+/tm1Asim^* (n = 10) and *Otp^+/+^* (n = 10), (**d**) female *Otp^R108W/+^* (n = 17) and *Otp^+/+^* (n = 17), (**e)** male *Otp^+/tm1Asim^* (n = 12) and *Otp^+/+^* (n = 10) , **(f**) female *Otp^+/tm1Asim^* (n = 10) and *Otp^+/+^* (n = 10) mice, (**g**) *Otp^R108W/+^* (n = 17) and *Otp^+/+^* (n = 16-17), (**h**) male *Otp^+/tm1Asim^* (n = 12) and *Otp^+/+^* (n = 10), (**i**) female *Otp^+/tm1Asim^* (n = 10) and *Otp^+/+^* (n = 10), (**j**) *Otp^R108W/+^* (n = 17) and *Otp^+/+^* (n = 16-17), (**k**) male *Otp^+/tm1Asim^* (n = 12) and *Otp^+/+^* (n = 10), (**l**) female *Otp^+/tm1Asim^* (n = 10) and *Otp^+/+^* (n = 10), (**m**) female *Otp^R108W/+^* (n = 17) and *Otp^+/+^* (n = 16), (**p**) female *Otp^R108W/+^* (n = 11) and *Otp^+/+^* (n = 9), (**q**) male *Otp^+/tm1Asim^* (n = 10) and *Otp^+/+^* (n = 7), (**r**) female *Otp^+/tm1Asim^* (n = 10) and *Otp^+/+^* (n = 6)

**Statistical Analysis**

For (**a-c**) the AUC was calculated individually baselined to 3 (**a**) or 4 (**b,c**) weeks of age showing increased AUC in heterozygotes p=<0.0001 for each. Differences in AUCs and individual timepoints were tested with an unpaired Mann Whitney 2-tailed t-test.

For (**d-f**) AUCs were calculated using individual 4 week baselines showing increased AUC in heterozygotes p=<0.001. Differences in AUCs and individual timepoints were tested with an unpaired Mann Whitney 2-tailed t-test.

For (**g**) an unpaired Mann-Whitney two-tailed t-test was used within age points and a Wilcoxon 2-tailed paired t test between time-points for the same genotype. For (**h**) male data was tested with a two-tailed unpaired t-test with Welch’s correction at individual timepoints and a two-tailed paired t-test with Welch’s correction between the same genotypes at different timepoints. For (**i**) data was tested with a two tailed unpaired t-test with Welch’s correction at individual timepoints and a Wilcoxon matched-pairs signed rank test between the same genotypes at different timepoints.

For (**j**) an unpaired Mann-Whitney two-tailed t-test was used within age points and a Wilcoxon 2-tailed paired t test between time-points for the same genotype. For (**k and l**) individual timepoints were tested with a unpaired Mann-Whitney 2-tailed t test at individual timepoints and a Wilcoxon matched-pairs signed rank test between the same genotypes at different timepoints.

For (**m,n,o**) AUCs were calculated using individual t=0 baselines and was increased in *Otp^R108W/+^* heterozygotes p=<0.0001 and heterozygote knockouts (male) p=<0.0015 and (female) p=<0.0052. Differences in AUCs and individual timepoints were tested with a pairwise Mann Whitney 2-tailed t-test.

For (**p**) AUC was calculated baselined to 0 and was increased in heterozygotes p=<0.0012. Differences in AUCs and individual timepoints were tested with an unpaired Mann Whitney 2-tailed t-test. For (**q and r**) Data analyzed by two-way ANOVA without repeated measures. Four *Otp^+/tm1Asim^* and three *Otp^+/+^*females had to be removed from the test at or after 60 minutes, or at 90 minutes respectively because their blood glucose level had dropped below the acceptable range. Similarly, 1 wildtype male was removed at 90 minutes.


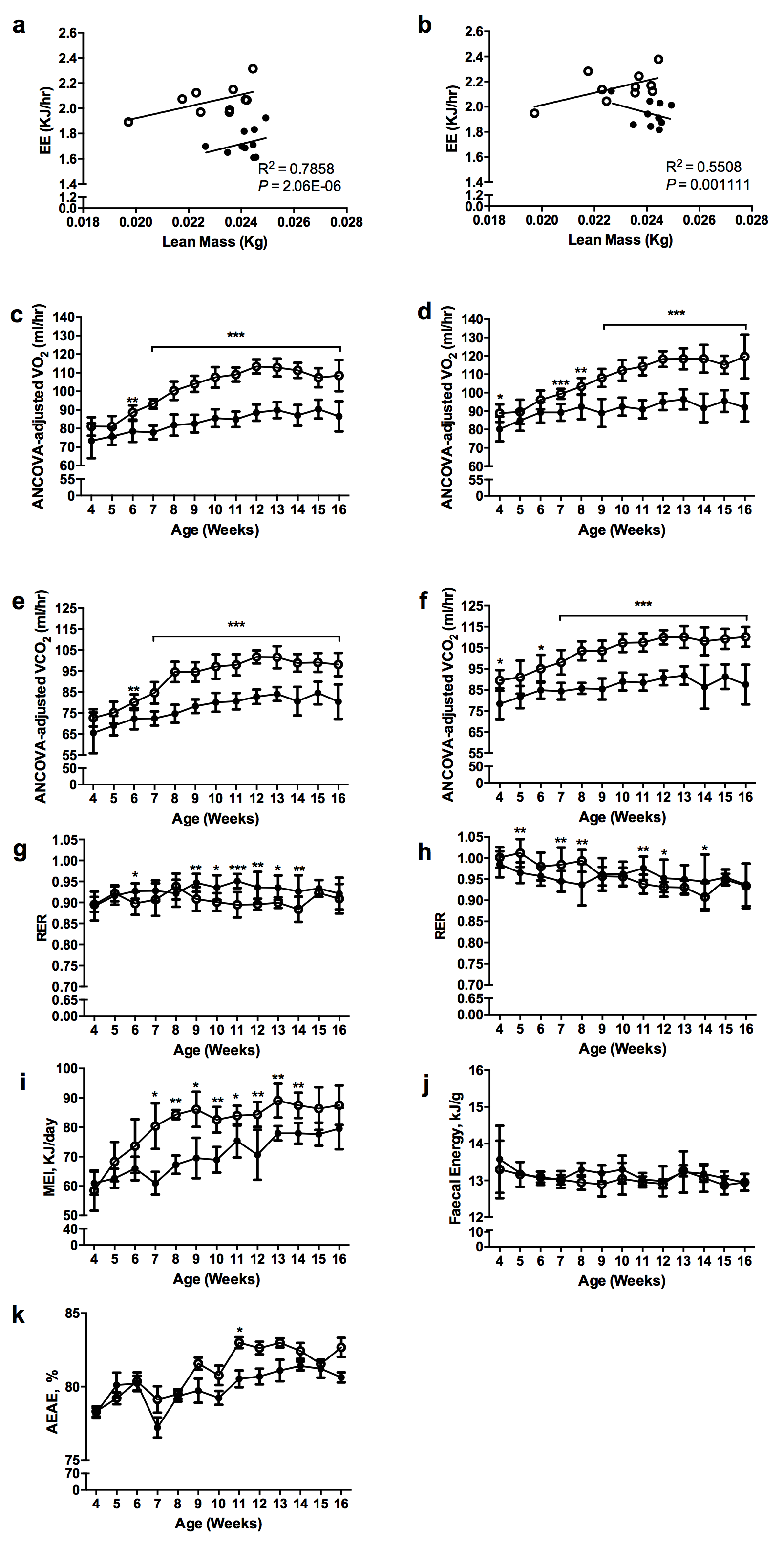


**Supplementary Figure 2. Related to Figure 2 g-i. Additional energy balance data for *Otp^R108W/+^* mice**

(**a** and **b**) Unadjusted Energy Expenditure regressed against lean mass at 8 weeks of age (10 mice in each group). (**c-h**) Weekly indirect calorimetry between 4 and 16 weeks of age collected over a 21 hour period. *Otp^R108W/+^* (n = 10) and *Otp^+/+^* (n = 8-10) male mice. Oxygen consumption (VO_2_), carbon dioxide production (VCO_2_) and RER during the light period (**a,c,e,g**) and dark (**b,d,f,h**) period respectively. *Otp^R108W/+^* mice had significantly increased VO_2_ (**c**) and VCO_2_ (**e**) from 6 weeks in the light period. VO_2_ was significantly increased in the dark period at 4 and from 7 weeks in *Otp^R108W/+^* mice (**d**). VCO_2_ was significantly increased in the dark period at 4 and from 6 weeks in *Otp^R108W/+^* mice (**f**). Respiratory exchange ratio (RER) during the light period (**g**) and dark (**h**) period respectively, was calculated as ratio of VO_2_ and VCO_2_. *Otp^R108W/+^* mice had significantly decreased RER at 6, 9, 10, 11, 12, 13 and 14 weeks of age in the light and significantly increased RER at 5, 7, 8, 11, 12 and 14 weeks in the dark period. VO_2_ and VCO_2_ values were adjusted for variation in lean mass using multiple linear regression analysis (ANCOVA). (**i**) Weekly metabolisable energy intake (MEI) of male *Otp^R108W/+^* and *Otp^+/+^* mice from 7 to 14 weeks of age, for both *Otp^+/+^* and *Otp^R108W/+^* (**j**) Faecal energy content of male *Otp^R108W/+^* and *Otp^+/+^*. Faecal energy was determined by burning ~ 1 g collected over a 24 hour period from every cage in a bomb calorimeter. Faecal energy multiplied by faecal weight per mouse gave a value for gross energy output. There were no significant differences between the genotypes. (**k**) Apparent energy assimilation efficiency (AEAE) of male *Otp^+/+^* and *Otp^R108W/+^* mice. This was the ratio of daily energy intake over metabolisable energy intake as a percentage. AEAE indicated the efficiency of the mice to extract energy from their chow. *Otp^R108W/+^* mice had significantly increased AEAE at 11 weeks of age only. For **g**, **h** and **I** values are calculated from the average of 5 cages each housing two mice of the same sex and genotype.

*Otp^R108W/+^* are open symbols and *Otp^+/+^* are filled symbols.

**Statistical analysis**

For (**a** and **b**) was analysed by multiple linear regression. For **(c to f**) analysis was by ANCOVA. For (**g and h**) an unpaired Mann Whitney 2-tailed t-test was used showing significantly elevated RER in heterozygotes at a number of timepoints in both light and dark phases. For **(i**) AUCs were calculated using individual 4-week baselines and was increased in heterozygotes p=<0.0079. Differences in AUCs and individual timepoints were tested with an unpaired Mann Whitney 2-tailed t-test showing increased MEI from 7-14 weeks in heterozygotes. * *P*<0.05, ** *P*<0.01, *** *P*<0.001. For (**j**) the curves overlapped and therefore we compared Wildtype (n=64) to heterozygotes (n=65) as a single group across the timecourse using a Mann-Whitney 2-tailed t test and found slightly less faecal energy (-0.093kj/) in heterozygotes (p=0.0315). For (**k**) data were analysed by 2-way ANOVA with Bonferroni post tests and timepoints were not significantly different apart from 11 weeks in AEE %.


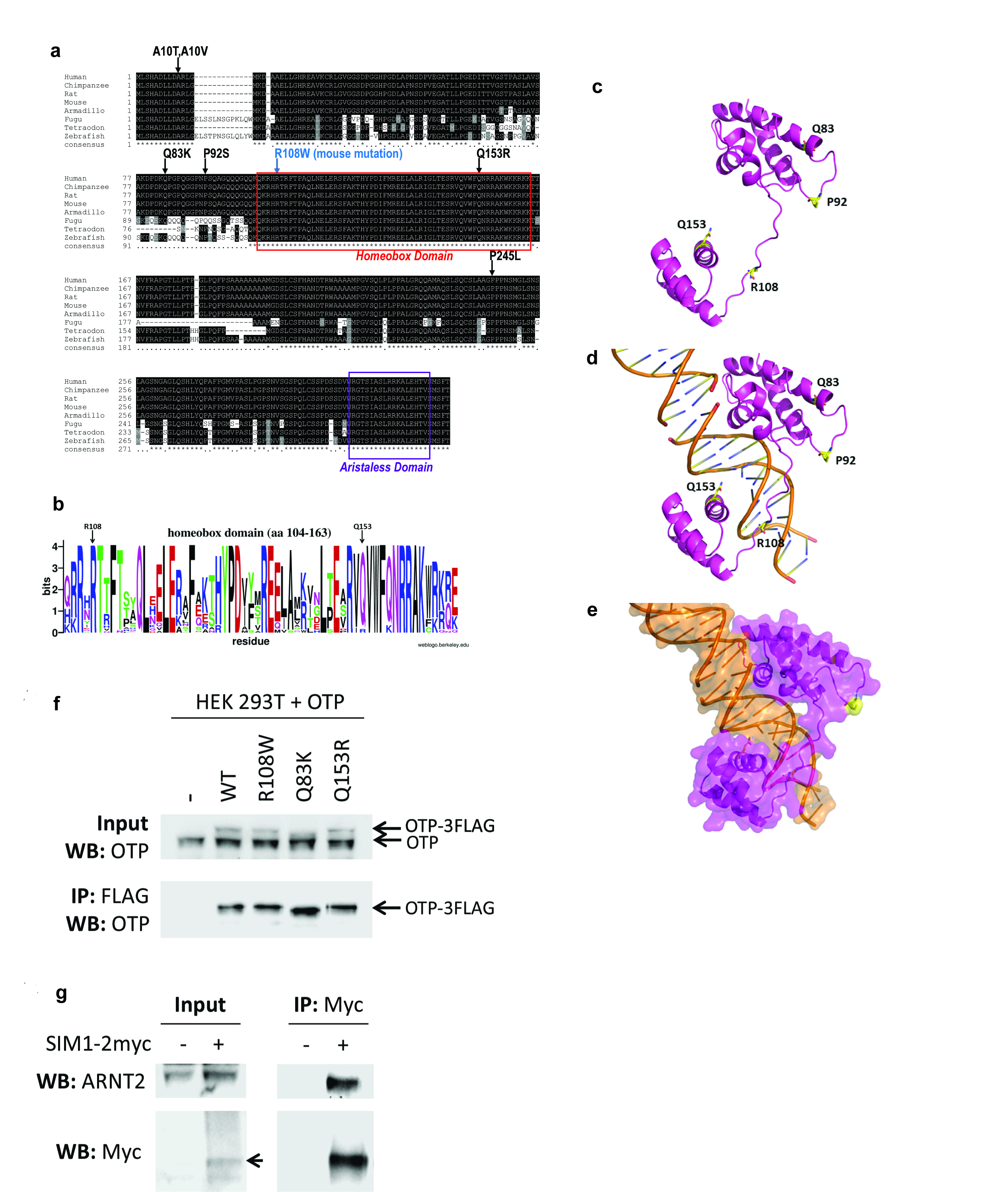


**Supplementary Figure 3. Related to Fig 4. OTP is a highly conserved protein and there is a lack of dimerisation between OTP mutants and wildtype OTP.**

(**a**) Conservation of OTP protein across multiple species is shown and the position of genetic variants identified in this study, the homeodomain, and OAR/aristaless domain indicated. (**b**) Consensus sequence for the homeodomain. Location of two variants OtpR108W and OtpQ153R within the highly conserved DNA-binding structural motif are indicated.

(**c-e**) An OTP homology model. (**c**) Highlighted residues (yellow sticks) correspond to 4 of the 6 mutant variants. R108 and Q153 are highly conserved and form a crucial part of the DNA-binding motif in OTP. (**d**) Molecular docking pose of OTP wild type homology model (residues 13-161) docked onto a fragment of DNA. (**e**) Surface representation of the molecular docking showing Q153 docks into the major groove, while R108 docks into the minor groove effectively wrapping itself around the DNA to form a stable complex.

**(f-g)** Lack of dimerisation between OTP mutants and WT OTP. Binding of Q153R to a palindromic sequence, but not a single site, raises the question of whether or not OTP dimers may exist in solution. If so, non-DNA binding mutants such as R108W may function in a dominant negative fashion by sequestering WT OTP. We therefore performed co-immunoprecipitations using extracts from cells expressing tagged variants of OTP and non-tagged wt OTP. However, we failed to see any evidence of dimerisation using conditions which maintained the dimeric SIM1/ARNT2 transcription factor. This suggests that OTP monomers co-operatively interact on palindromic DNA sequences, consistent with previous studies showing co-operative DNA binding by other homeodomain proteins[^24^](#_ENREF_24). (**f**) Whole cell extracts from HEK293T cells transiently transfected with WT or variant OTP-3xFlag expression vectors, plus non-tagged WT OTP, were subjected to immunoprecipitation with anti-Flag resin as previously described[^25^](#_ENREF_25). Input extracts (10%) and immunoprecipitates were subjected to Western blot analysis using 1/1000 dilution of anti-OTP polyclonal Ab (GeneTex); (**g**) Positive control co-immunoprecipitations showing dimerization between SIM1myc and ARNT2.


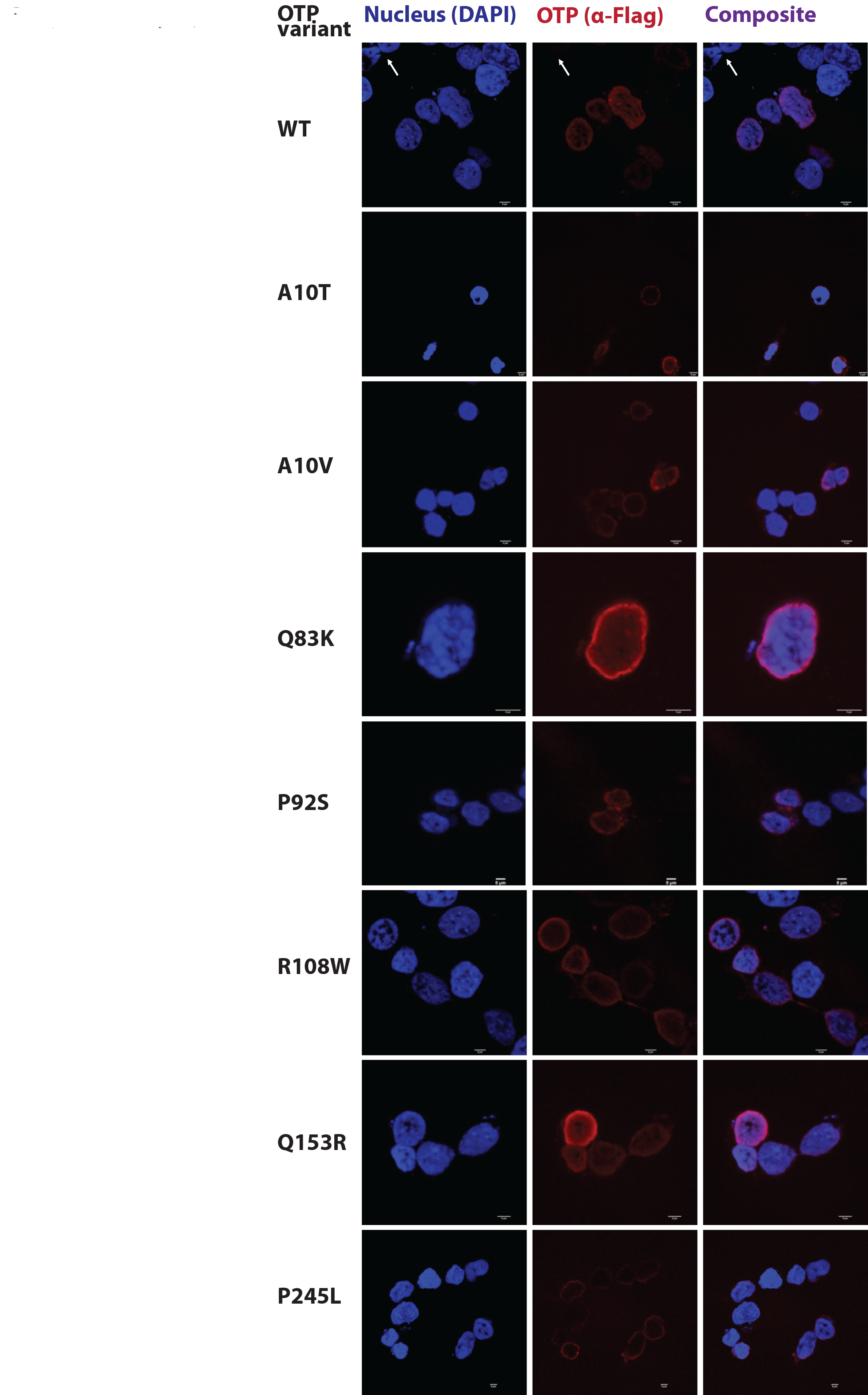


**Supplementary Figure 4. Related to Figure 4. Intracellular localisation of OTP mutants.**

HEK 293T cells transiently expressing Flag-tagged WT or mutant OTP were imaged using confocal microscopy. Scale bars: 5 μm. WT OTP and all OTP mutant proteins exhibited similar nuclear localisation pattern. Non-transfected cells are indicated with arrows, demonstrating lack of non-specific background staining.
