# Supplementary material for: Disruption of the homeodomain transcription factor orthopedia homeobox (Otp) is associated with obesity and anxiety
Source: Mol Metab. 2017 Aug 24;6(11):1419–28. doi: 10.1016/j.molmet.2017.08.006 (PMC5681237; doi:10.1016/j.molmet.2017.08.006)
Supplement: Supplementary file 3 [file mmc3.docx]

**Appendix C**

Disruption of the homeodomain transcription factor orthopedia homeobox (*Otp*) is associated with obesity and anxiety

Moir *et al.*

**Supplementary Tables**

**Table S1. *Otp^R108W/R108W^* mice die before weaning.**

|  | **P0/P1** | | **Weaning (P21)** | |
| --- | --- | --- | --- | --- |
|  | **Observed** | **Expected** | **Observed** | **Expected** |
| ***Otp^+/+^*** | 7 | 6.5 | 8 | 5.5 |
| ***Otp^R108W/+^*** | 11  (1 dead) | 13 | 14 | 11 |
| ***Otp^R108W/R108W^*** | 8  (2 dead) | 6.5 | 0 | 5.5 |
| **Total** | 26 | 26 | 22 | 22 |

Mortality rates of *Otp^+/+^*, *Otp^R108W/+^* and *Otp^R108W/R108W^* mice at birth (P0/P1) and weaning (P21). The numbers of pups of each genotype observed at birth (alive or dead) and the number expected by Mendelian ratio was not significantly different, P=0.707, X^2^ =0.692 with 2 degrees of freedom. In contrast no homozygous pups survived to weaning p=0.024, X^2^ 7.454 with 2 degrees of freedom.

**Table S2**. ***Otp^R108W/+^* show increased fat depot weights and reduced bone mineral density**

|  |  | ***Otp^+/+^*** | ***Otp^R108W/+^*** | ***P*^*^** |
| --- | --- | --- | --- | --- |
| **Gonadal fat pad weight (g) - terminal** | Male | 1.149 ± 0.06751, n=11 | 1.417 ± 0.07503, n=7 | **0.0189** |
|  | Female | 1.036 ± 0.1613, n=11 | 1.912 ± 0.1057, n=11 | **0.0003** |
| **Subcutaneous fat pad weight (g) - terminal** | Male | 0.3170 ± 0.01428, n=11 | 0.7409 ± 0.06272, n=7 | **0.0004** |
|  | Female | 0.5897 ± 0.05358, n=11 | 0.8218 ± 0.06805, n=11 | **0.0148** |
| **Bone Mineral Density (BMD, g/cm^2^) - 15-16 weeks of age** | Male | 0.06384 ± 0.0006229, n=10 | 0.0606 ± 0.0007803, n=9 | **0.0052** |
|  | Female | 0.06608 ± 0.0008597, n=10 | 0.06134 ± 0.001247, n=10 | **0.0065** |
| **Bone Mineral Content (BMC, g) - 15-16 weeks of age** | Male | 0.5957 ± 0.01077, n=10 | 0.5814 ± 0.01737, n=9 | 0.4972 |
|  | Female | 0.6264 ± 0.01153, n=10 | 0.6332 ± 0.01806, n=10 | 0.7553 |

Mean +/- standard error, ^*^unpaired two-tailed t-test’s with Welch’s correction

**Table S3. Compound heterozygous *Otp^R108W/tm1Asim^* mice do not survive to weaning.**

|  | **P0/P1** | | **Weaning (P21)** | |
| --- | --- | --- | --- | --- |
|  | **Observed** | **Expected** | **Observed** | **Expected** |
| ***Otp^+/+^*** | 4 | 5.25 | 11 | 8 |
| ***Otp^R108W/+^*** | 6 | 5.25 | 8 | 8 |
| ***Otp^+/tm1Asim^*** | 7 | 5.25 | 13 | 8 |
| ***Otp^R108W/tm1Asim^*** | 4 | 5.25 | 0 | 8 |
| **Total** | 21 | 21 | 32 | 32 |

Numbers of Offspring Produced from *Otp^R108W/+^* *Otp^+/tm1Asim^* Intercrossed on a C57BL/6J background. The numbers of pups of each genotype observed at birth (alive or dead) and the number expected by Mendelian ratio was not significantly different, P=0.733, X^2^ =1.29 with 3 degrees of freedom. In contrast no compound heterozygous pups survived to weaning, p=0.007, X^2^ =12.25 with 3 degrees of freedom.

**Table S4. Reduced T4 thyroid hormone plasma concentrations in *Otp^R108W/+^* mice**

|  | ***Otp^+/+^*** | ***Otp^R108W/+^*** | **p^*^** |
| --- | --- | --- | --- |
| **T3 (ng/mL)** | 1.57 ± 0.22, n=7 | 1.36 ± 0.19, n=7 | 0.0758 |
| **T4 (μg/dL)** | 4.85 ± 0.14, n=7 | 4.11 ± 0.16, n=7 | **0.0047** |

Measurement of plasma T3 and T4 concentrations in *Otp^+/+^* and *Otp^R108W/+^* . Analysed with an unpaired two-tailed t-test with Welch’s correction.

**Table S5. Elevated male urinary corticosterone concentrations in OtpR108W/+ Mice**

|  | **Corticosterone ng/ml** | |  |
| --- | --- | --- | --- |
|  | ***Otp^+/+^*** | ***Otp^R108W/+^*** | ***P^*^*** |
| **4 weeks** | 69.74 ± 14.50, n=4 | 322.8 ± 98.28, n=6 | 0.0055 |
| **6-10 weeks** | 71.27 ± 12.08, n=18 | 111.3 ± 16.29, n=10 | 0.0084 |
| **17-21 weeks** | 141.8 ± 16.50, n=10 | 529.1 ± 90.48, n=7 | < 0.0001 |

* The data was log transformed (y=log(y)) to normalise the distribution (data not shown) and then tested with a two-tailed unpaired t-test with Welch’s correction at individual timepoints

**Table S6. Genetic association of variants in OTP in Obesity cases and UK10K controls.**

|  | **Number of Rare Alleles** | |  |  |  |  |  |
| --- | --- | --- | --- | --- | --- | --- | --- |
| **Test** | **SCOOP Targeted (N = 1811)** | **SCOOP WES (N = 737)** | **Controls WES (N = 1117)** | **OR** | **Adj. OR*** | **p-value**  **SKATO** | **p-value BURDEN** |
| **Only p.Q83K (rs143794465)** | **19** | **12** | **9** | **1.516** | **NA** | **NA** | **0.35** |
| **4 Rare Functional variants** | **36** | **18** | **16** | **1.482** | **NA** | **0.23** | **0.15** |

Association of rare and novel functional variants in OTP (see Methods). *adjusted OR - odds ratio calculated after adding 0.5 to the number of alleles in each cell of the two-by-two table. NA=not applicable.

**Table S7. Phenotypes of *OTP* mutation carriers.**

|  | **Gender** | **Age**  **(years)** | **BMI sds** | **Height sds** | **Other conditions** |
| --- | --- | --- | --- | --- | --- |
| **A10T** | M | 15 | 3.8 | 1.3 | Raised TSH |
| **A10V** | F | 12 | 3.8 | 0.9 |  |
| **Q83K** | M | 13 | 2.5 | -2.5 | GH deficiency |
| **Q83K** | F | 13 | 4.0 | 0.8 | Developmental delay; Aspergers syndrome |
| **P92S** | M | 15 | 3.7 | -0.1 |  |
| **Q153R** | M | 11 | 4.3 | 1.0 | inattentive type attention deficit disorder; acanthosis nigricans |
| **P245L** | M | 13 | 4.1 | 1.1 |  |

M=male; F=female. Body mass index (BMI) and height standard deviations scores (sds) were calculated using UK reference data. TSH=thyroid stimulating hormone; GH=growth hormone.
